# Long-Term Biogas Production from Glycolate by Diverse and Highly Dynamic Communities

**DOI:** 10.3390/microorganisms6040103

**Published:** 2018-10-04

**Authors:** Susanne Günther, Daniela Becker, Thomas Hübschmann, Susann Reinert, Sabine Kleinsteuber, Susann Müller, Christian Wilhelm

**Affiliations:** 1 UFZ-Helmholtz Centre for Environmental Research, Department of Environmental Microbiology, Permoserstraße 15, 04318 Leipzig, Germany; susann.guenther@ufz.de (S.G.); daniela.taraba@ufz.de (D.B.); thomas.huebschmann@ufz.de (T.H.); sabine.kleinsteuber@ufz.de (S.K.); 2 Institute of Biology, University of Leipzig, Johannisallee 21–23, 04103 Leipzig, Germany; susann.reinert@gmx.de (S.R.); cwilhelm@rz.uni-leipzig.de (C.W.)

**Keywords:** microbial community dynamics, microbial flow cytometry, anaerobic digestion, glycolate-fermenting bacteria, methanogenic archaea

## Abstract

Generating chemical energy carriers and bulk chemicals from solar energy by microbial metabolic capacities is a promising technology. In this long-term study of over 500 days, methane was produced by a microbial community that was fed by the mono-substrate glycolate, which was derived from engineered algae. The microbial community structure was measured on the single cell level using flow cytometry. Abiotic and operational reactor parameters were analyzed in parallel. The R-based tool flowCyBar facilitated visualization of community dynamics and indicated sub-communities involved in glycolate fermentation and methanogenesis. Cell sorting and amplicon sequencing of 16S rRNA and *mcrA* genes were used to identify the key organisms involved in the anaerobic conversion process. The microbial community allowed a constant fermentation, although it was sensitive to high glycolate concentrations in the feed. A linear correlation between glycolate loading rate and biogas amount was observed (R^2^ = 0.99) for glycolate loading rates up to 1.81 g L^−1^ day^−1^ with a maximum in biogas amount of 3635 mL day^−1^ encompassing 45% methane. The cytometric diversity remained high during the whole cultivation period. The dominating bacterial genera were *Syntrophobotulus*, Clostridia genus B55_F, *Aminobacterium,* and *Petrimonas*. Methanogenesis was almost exclusively performed by the hydrogenotrophic genus *Methanobacterium*.

## 1. Introduction

The replacement of fossil fuels by renewable energy sources is essential to mitigate global warming. The conversion of biomass into gaseous or liquid biofuels is generally considered sustainable [1], but the energy conversion efficiency is low [2]. However, most biofuels are currently produced from energy crops grown on valuable agricultural land, thus competing with food and feed production [3]. While microalgae are an alternative feedstock for biofuel production [4] their utilization includes high costs due to the investment for the photobioreactors and operation for stirring, harvesting or transport to biorefinery plants [5]. A new approach [6,7] proposed a concept in which photosynthetic energy is mainly conserved in the form of glycolate by a controlled balance between carboxylation and oxygenation by the ribulose-1,5-bisphosphate carboxylase/oxygenase (RubisCO) in *Chlamydomonas reinhardtii.* The produced glycolate is efficiently excreted by the microalgae because its metabolization via the C2 cycle is blocked [7]. The concept minimizes both metabolic and economic costs of glycolate production. In the present study, we investigated whether the excreted glycolate can be efficiently converted to methane by a subsequent anaerobic digestion process. Consortia of syntrophic bacteria and methanogenic archaea that can convert glycolate to methane have been already described [8,9]. While several aerobic degradation pathways of glycolate are well known such as the dicarboxylic pathway in *Escherichia coli* [10,11], the glycerate pathway in *E. coli* [12], *Pseudomonas* sp. [13] and *Azotobacter chroococcum* [14], and the β-hydroxyaspartate pathway in *Micrococcus denitrificans* [15], the metabolization of glycolate under anaerobic conditions is less well explored. Only a few isolates have been described for anaerobic glycolate conversion such as *Desulfofustis glycolicus* and *Syntrophobotulus glycolicus* [16], *Moorella* sp. strain HUC22-1 [17], *Moorella thermoacetica* [18], and Lachnospiraceae strain 19gly4 [19], which use the malyl-CoA-pathway [20]. Some of the fermentation products, i.e., hydrogen and carbon dioxide, formate or actetate, can be directly converted to methane.

The set-up proposed in this study relies on glycolate as mono-substrate for methane production [6,7,8,9]. Other mono-substrates such as acetate [21], butyrate [22], propionate [23], and glucose [24] were already shown to be suitable substrates for continuous methane production. However, glycolate could be problematic for the process due to the potentially small group of anaerobic glycolate utilizers. Moreover, in contrast to the anaerobic oxidation of propionate or butyrate, which is only possible by syntrophic interaction of proton-reducing bacteria and hydrogen-scavengers such as hydrogenotrophic methanogens, glycolate can be expected to be fermented directly to acetate by homoacetogens such as *Moorella* via the Wood-Ljungdahl pathway [17] or to other fermentation products such as succinate and acetate by single strains such as Lachnospiraceae strain 19gly4 [19] and thus its degradation to acetate does not necessarily require the involvement of methanogens. In that case, conversion to methane would rely on the presence of acetoclastic methanogens. However, glycolate can also be exploited by hydrogenotrophic methanogens together with syntrophic proton-reducing bacteria that are needed to perform the oxidation of glycolate to glyoxylate and further to carbon dioxide and hydrogen [9].

Usually, biogas is produced by natural microbial communities from complex substrates. These microbial systems behave dynamically and convey short reaction times to external changes [25]. Molecular tools are typically used to analyze the microbial community composition [26,27]. However, these methods have limitations for routine applications especially when fast dynamics, which require dense sampling procedures over longer time scales, are expected. Missing sampling points can aggravate for instance association analyses by using *Cytoscape* [28] and *CoNet* [29], which help find functional key organisms in microbial communities. Flow cytometry is an alternative approach, especially since bioinformatic tools are now available that enable the interpretation of fast shifts of microbial community structures using *flowCHIC* [30] and *flowCyBar* [31]. These tools grant the accurate quantitative analysis of cell abundance variation and allow via correlation analyses with abiotic data to attribute metabolic functions to specific sub-communities [32,33].

The aim of this study was to challenge an anaerobic digester community to continuously convert the mono-substrate glycolate to methane at high turnover rates and over long time periods. Possible positive or negative influences of reactor parameters on biogas production and the function of microbial key players that contribute to either glycolate fermentation or methanogenesis were of interest to obtain means for process control.

## 2. Materials and Methods

### 2.1. Digester Configuration and Operational Parameters

The biogas reactor had a working volume of 3.5 L and was operated at 37 °C (Supplemental Figure S1). Temperature was controlled by a water bath (E100, Lauda, Germany). A pump (101U, Watson-Marlow GmbH, Rommerskirchen, Germany) controlled the feeding via a time switch (25750, REV-Ritter GmbH, Eisenach, Germany), thereby adjusting hydraulic retention time (HRT) and glycolate loading rate (GLR). Stirring was operated during feeding (60 rpm, Modelcraft RB-35, Conrad, Germany) and 30 s prior to sampling. The feed solution contained, besides glycolate, salts, trace elements, and vitamins (Supplemental Table S2). Sampling was done via a sampling tube and a gas collection tube (350 mL, VWR International GmbH, Langenfeld, Germany). The biogas reactor was operated as a single approach, inoculated with digestate from a full-scale agricultural biogas plant (Rackwitz, Germany) fed with cattle manure and maize silage. Before starting the process, the reactor was flushed with nitrogen to establish anoxic conditions. The anaerobic digester was adapted to the feed solution and operated over 2.5 years before perturbations were artificially invoked. 

### 2.2. Analytical Methods

The pH was determined and the VOA/TIC ratio was calculated as ratio between volatile organic acids (VOA) and total inorganic carbon (TIC) [34] measured by titration (Knick Portamess 913, WTW, Weilheim, Germany) of a 1:10 diluted reactor sample with 5 mM H_2_SO_4_ (Roth, Bavaria, Germany). The concentrations of acetic, propionic and glyoxylic acid in the reactor samples were determined by using a 10AVP HPLC System (Shimadzu, Duisburg, Germany) equipped with a Rezex ROA-Organic Acid H^+^ (8%) 300 × 7.8 mm column (Phenomenex, Aschaffenburg, Germany) and a RI detector (RID-10A, Shimadzu, Duisburg, Germany) at 70 °C. As a mobile phase, 5 mM H_2_SO_4_ (Roth, Bavaria, Germany) was used at a flow rate of 0.6 mL min^−1^. The reactor sample was filtered before measurement (syringe filter, 0.2 µm, VWR, Langenfeld, Germany). The daily biogas amount (mL day^−1^) was monitored using a milligascounter (MGC-1, Ritter, Germany) and normalized to standard conditions. The productivity of biogas formation was calculated in mL per gram glycolate per day. Biogas composition was measured by gas chromatography (Chrompack CP 9001, Shimadzu, Duisburg, Germany) with a CarboPlot P7 25 × 0.53 column and a thermal conductivity detector (Micro TCD 2045 Detector, Chrompack, Shimadzu, Duisburg, Germany). Argon served as carrier gas (1.3 mL min^−1^). Before measurement, the gas syringe (Clexane, 1 mL) was flushed with argon to exclude air contaminations. All analytical data are listed in Supplemental Table S3. 

### 2.3. Flow Cytometry and Cell Sorting

Samples were harvested, stabilized with 2% paraformaldehyde (Serva, Heidelberg, Germany), vortexed and incubated for 30 min at room temperature (RT). Thereafter, cells were washed in phosphate-buffered saline (PBS, S2) at 3,200× *g* for 10 min, 4 °C, and the pellet resuspended in 70% ethanol (Roth, Bavaria, Germany) and stored at −20 °C [33]. For cytometric analysis, the cells were washed as before and adjusted to an optical density of 0.035 (dλ700 nm = 0.5 cm) with 400 mM Na_2_HPO_4_, pH 7.0 and re-suspended in 0.5 mL of a 11 mM citric acid and 0.8 mM Tween 20 solution. The sample was sonicated (Sonorex Digitec, Bandelin Electronic GmbH Co. KG, Berlin, Germany) for 5 min and incubated for further 25 min. After another washing step as before, the cell pellets were stained with 1 mL 1 μM 4′,6-diamidino-2′-phenylindole (DAPI, Sigma, Roedermark, Germany) in 400 mM Na_2_HPO_4_, pH 7.0 for 3 h at RT, and vortexed. Samples were filtered through 50 μm CellTrics^®^ (Partec, Hamburg, Germany) prior to cytometric measurement. Fluorescence beads (0.5 μm Fluoresbrite carboxy BB microspheres (Polysciences, Niles, IL, USA) and 1 μm Polymer microspheres 350/530 (ThermoFischer Scientific GmbH, Dreieich, Germany) were added to the samples as internal standard. For all measurements, at least 250,000 stained cells were recorded. The files are available at the FlowRepository (https://flowrepository.org/id/RvFrPg2aOOvne2qTF5ksrMT6IwQciR66Iel7bsddUqIH05JyhsWnGUPSsOzondKk).

A MoFlo cell sorter (DakoCytomation, Santa Clara, CA, USA) equipped with a 488 nm argon laser (400 mW) and a ML-UV laser (333–365 nm, 100 mW) was used for the cytometric measurements. Forward scatter (FSC, 488/10) and side scatter (SSC, trigger signal, 488/10) were used for the analysis of intrinsic cell parameters. The ML-UV excitation was used to measure DAPI fluorescence (450/65). Fluorescent beads (2 μm, yellow-green FluoSpheres 505/515 and 1 μm, blue Fluo-Spheres 350/440 (Molecular Probes, Eugene, OR, USA) were used for the instrument adjustment. Cytometric data were analyzed with Summit V 4.3 (Beckman-Coulter, Brea, CA, USA). The cell-sorting procedure was done according to the protocol by Koch et al. [35]. A representative number of samples were chosen under the premise of comprising 500,000 cells per gate, which were selected for sorting. The most accurate sort mode of the MoFlo (single and one-drop mode: highest purity 99%) at a rate not higher than 2500 events/s was adjusted. After sorting, cells were harvested by centrifugation (20,000× *g*, 4 °C, 25 min), and the pellet was frozen at -20 °C for later DNA extraction and Illumina MiSeq sequencing. For detailed information regarding the chosen samples and gates, see Supplemental Table S4.

### 2.4. Microbial Community Analysis by Amplicon Sequencing

DNA from sorted cells was extracted as described in Supplemental Table S5. Bacterial community compositions of sub-communities sorted from samples on days 495 (G1, G2, G26) and 508 (G3, G6, G8, G25) were analyzed by amplicon sequencing of 16S rRNA genes. The V3–V4 region of the 16S rRNA genes was amplified using the primer pair Pro341F and Pro805R [36]. Methanogenic archaea in the sub-communities were analyzed by amplicon sequencing of *mcrA* genes using the primers described by Luton et al. [37]. Amplicon sequencing was performed on the Illumina MiSeq platform (V3, 2 × 300 bp), for details see Supplemental Table S5. Primer and barcode clipping was done using the fastX toolkit [38]. Paired-end reads were merged using PEAR 0.9.10 [39]. Quality filtering of the merged sequences and clustering into operational taxonomic units (OTUs) at 97% or 95% similarity threshold for 16S rRNA genes and *mcrA* genes, respectively, were done with the QIIME 1.9.1 Virtual Box release [40]. OTU picking at a minimum cluster size of 5 and removal of chimeric sequences by de novo check were done using USEARCH [41]. For taxonomic assignment using the RDP Classifier 2.2 [42] at a confidence threshold of 0.8, the MiDAS 2.1 database [43] was used for the 16S rRNA OTUs, while the *mcrA* OTUs were assigned to a customized database created from the RDP FunGene [44]. Raw de-multiplexed sequence data were deposited at EMBL European Nucleotide Archive (ENA) under accession number: PRJEB27800.

### 2.5. Biostatistics

Sub-communities of stained cells were marked with gates using a facilitated gating procedure [45] and their abundance variation was followed over time. Statistical analyses were performed with R 3.4.4. Community and correlation analyses were either done using the package “flowCyBar” [31] or the package “Hmisc” [46]. For correlation analysis the Spearman’s rank order correlation coefficient rho was used in a moving window approach [47,48] encompassing 20 time points. *p*-values were corrected using the method of Benjamini and Hochberg [49]. The resulting data were visualized as functional heat maps specifying the direction and strength of the correlation data as color code [31,33].

## 3. Results

### 3.1. Glycolate as Sole Carbon Source for Biogas Production

**Figure 1:**
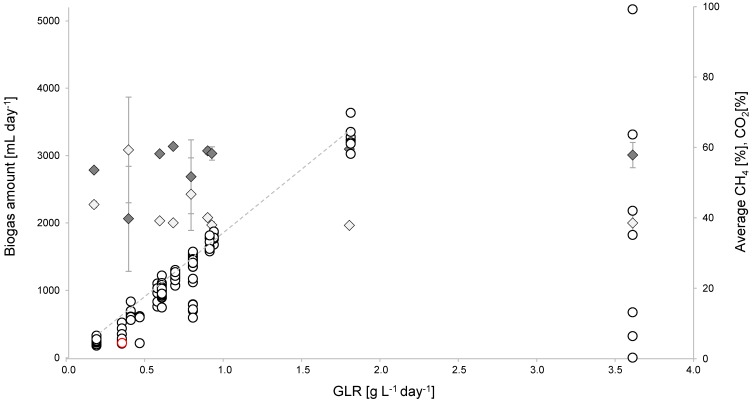
Daily biogas amount (open circles) depending on the glycolate loading rate (GLR). In addition, the methane (light grey rhombs) and carbon dioxide (dark grey rhombs) concentrations [%] in the biogas are given with respective standard deviation for each GLR. A linear correlation (R^2^ = 0.99) between GLR and biogas amount is indicated by the grey trend curve (y = 1906.1x − 57.941). The red colored circle indicates the restart of the reactor using a back-up inoculum from the reactor.

A microbial community originating from a conventional full-scale biogas reactor fed with maize silage and cow manure was cultivated for 526 days in a 3.5 L bench-top reactor on the mono-substrate glycolate. This long-term process was designed to determine the maximum biogas production capacity on the mono-substrate glycolate by increasing loading rates. A linear correlation was observed between GLR and the daily biogas amount (R^2^ = 0.99, Figure 1) up to a GLR of 1.81 g L^−1^ day^−1^. A further increase in the GLR up to 3.6 g L^−1^ day^−1^ did not cause higher biogas production. This was due to a pH drop to 3.25 because the buffer capacity of the medium was exceeded by the acidity of glycolate leading to a breakdown of the process. Until then, the biogas composition was relatively constant and showed average values of about 41.28% ± 2.83% CH_4_ and 56.1% ± 2.87% CO_2_ (Figure 1). The process was restarted by using a back-up inoculum from the reactor itself (red circle, Figure 1), and the biogas amount slowly increased again parallel to an increasing GLR.

The glycolate feed solution had a pH of 3, which is also a typical feature of maize silage in conventional biogas plants. While it can be assumed that the carbonate buffer in the reactor is strong enough to balance the pH at the glycolate concentration used, the feed solution was additionally buffered and adjusted to a pH of 7 for about 20 days. Interestingly, the biogas composition changed to 54.38% ± 9.16% CH_4_ and 43.36% ± 7.33% CO_2_, but the biogas amount decreased during this period. Steady high biogas production was re-obtained after addition of pH-uncorrected glycolate feed. All raw data can be found in Supplemental Table S3a.

### 3.2. Microbial Community Dynamics on Glycolate as Mono-Substrate

**Figure 2:**
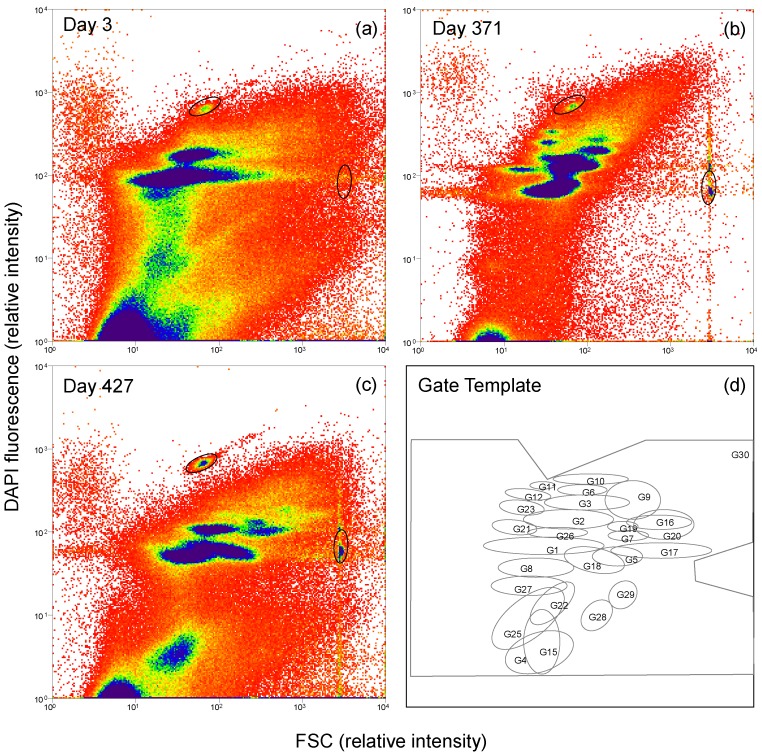
Microbial communities were analyzed using flow cytometry via the forward scatter signal (FSC) and DAPI staining of single cells. The varying structure of the community is exemplarily shown for three sampling days: day 3 (**a**), day 371 (**b**), and day 427 (**c**). Beads (0.5 and 1 µm, marked with ellipses) were used to align the 2D plots to each other. A gate template (**d**) was created to enable quantitative cell number determination in sub-communities over time.

**Figure 3:**
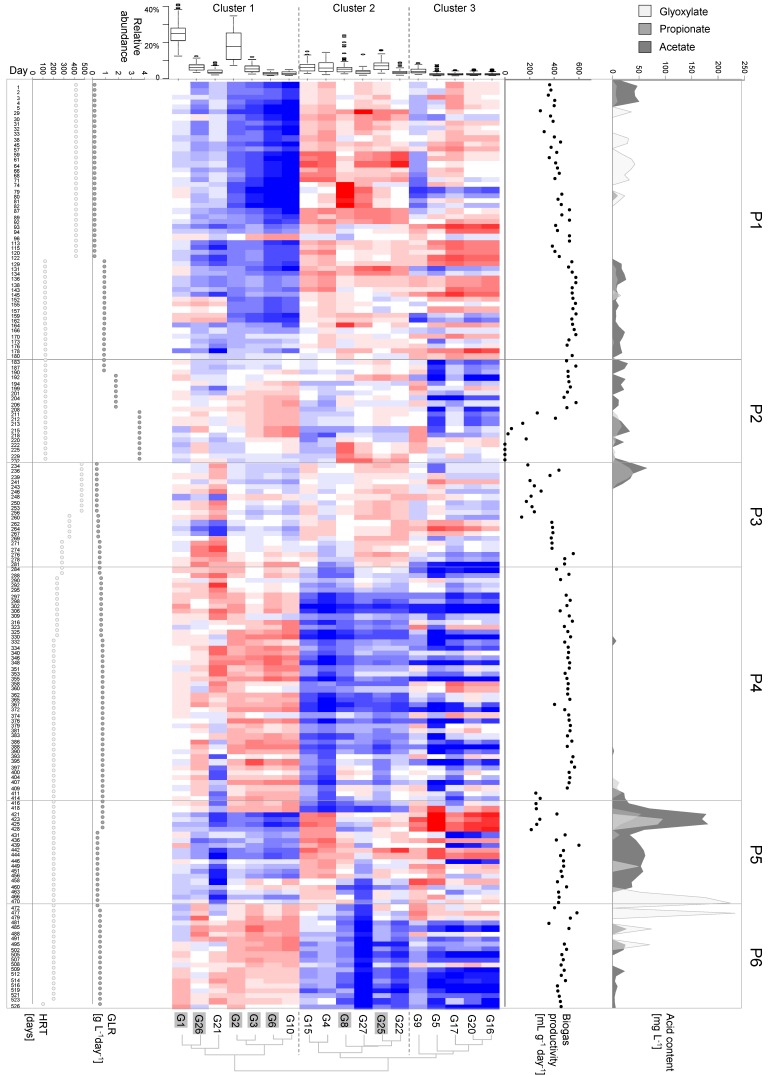
Shown from left to right are the hydraulic retention time (HRT [days]), the glycolate loading rate (GLR, [g L^−1^ day^−1^]), the cytometric barcode with boxplot (relative cell abundance per gate, %), biogas productivity [mL g^−1^ day^−1^], and acid concentrations [mg L^−1^] for acetate (dark grey), propionate (grey), and glyoxylate (light grey) over 526 days of sampling. For the cytometric barcode, mean normalized data were log transformed and ordered according to their Euclidean distance as calculated by flowCyBar. Sorted gates are marked with a grey background. Blue colors indicate a decrease below the mean of the cell number in the respective gate, red an increase, and white no change at all. Six different phases (P1–P6) were identified based on process parameters: P1: days 1–180, P2: days 183–232, P3: days 234–281, P4: days 284–414, P5: days 416–470, and P6: days 472–526. Due to their similarity in structure, three different cell clusters were recognized (clusters 1–3).

Changes in community structure on the cellular level were analyzed routinely using flow cytometry while changes in phylogenetic composition were detected exemplarily via cell sorting of specific sub-communities and amplicon sequencing of 16S rRNA and *mcrA* genes. By flow cytometry, 178 samples were analyzed and the bioinformatic evaluation was done by using the R package “*flowCyBar*”, which calculated cell abundance variations per each gate and histogram (all data in Supplemental Table S3b). For the cytometric dataset, 29 conclusive sub-communities and one master gate (G30, containing all cells: 100%) were defined, which were documented by gates in a designed gate template (Figure 2). Eleven sub-communities with average cell abundances below 1% were excluded from the analysis. Eighteen sub-communities had average cell abundances between 1% and 24% and covered on average 86.77% ± 6.11% of the cells of the master gate (Supplemental Table S3b). Sub-communities G1 (on average 24%) and G2 (on average 17%) had the highest cell abundances (Figure 3, boxplot). The community structure variations were finally correlated to the formation of metabolites such as biogas, acetate, glyoxylate, and propionate, which were measured in parallel to test for active functions of sub-communities.

The overview on the cytometric structure changes is given in Figure 3. Clusters of sub-communities with similarities in all cell abundance variations over the cultivation period were defined using Euclidean distance calculations thus defining cluster 1 with gates G1–G3, G6, G10, G21, and G26 (internal distance: 9.27 ± 1.8), cluster 2 with gates G4, G8, G15, G22, G25, and G27, (internal distance: 8.27 ± 1.2), and cluster 3 with gates G5, G9, G16, G17, G20 (internal distance: 9.61 ± 2.08). The average distance for all sub-communities was 15.2 ± 5.72 (all distances can be found in Supplemental Table S6). As could be anticipated from Figure 3, the three clusters comprised different parts of the microbial community. Cluster 1 was by far the largest cluster with 55.29% average abundance over the whole experiment. Cluster 2 was the second largest cluster with 21.66% and cluster 3 the smallest with 6.98% (Supplemental Table S7).

Phase 1, assigned to days 1 to 180, was characterized by comparatively low average mean values of cell numbers in cluster 1 (38.9%), while the cell numbers in the other clusters (2 and 3) showed abundances mainly above the average mean values of the respective gates (29.7% and 8.23%). During this phase the community maintained a constant structure and biogas productivity was stable and increased with the GLR. The intermediates glyoxylate (light grey) and acetate (dark grey) were found in considerable concentrations on almost all sampling days with the exception of days 87 to 129 (see Supplemental Table S3). Surprisingly, propionate (grey), a byproduct not predicted by the described anaerobic glycolate degrading pathways known up to now, was also detected on several days (e.g., days 134–145). At the end of phase 1, the structure of the community changed slowly, either due to the increased GLR or the decreased HRT because no other routinely measured operational parameter indicated a prospective influence on community structure variation.

On day 183 the community structure started to change more pronounced. In this second phase, cluster 2 and 3 sub-communities suddenly decreased in abundance (20.39% and 6.31%). At the end of this phase, a completely changed pattern was observed, which was accompanied by a drastic drop in the biogas productivity after day 206. The increase of the GLR from 1.81 to 3.61 g L^−1^ day^−1^ was possibly the key factor that led to reactor failure. The reactor acidified quickly after day 212, as was represented by an increase in VOA/TIC to 1.7; and at day 232 the pH reached 3.3 (see Supplemental Table S3a) and no biogas was produced after day 222.

Since a fast recovery of the acidified reactor was not anticipated in phase 3, the process was restarted from backup samples on day 234 with a low GLR of 0.34 g L^−1^ day^−1^ and a long hydraulic retention time of 470.4 days, both chosen similar to the original starting conditions. Indeed, the biogas productivity recovered to nearly phase 1 values within the following 30 days. Acids (acetate and propionate) were detected only from days 234 to 243 and not detectable afterwards. In addition, the community structure, shown by the distribution of cell abundances between sub-communities, was different in comparison to phase 1.

Phase 4 covered a long stable phase of 130 days until day 414, when the GLR was only slightly increased and the HRT decreased. The linear relationship between GLR and daily biogas amount was re-established as in phase 1. Until day 414 only samples taken on days 332 and 390 showed slightly elevated acetate concentrations. However, the community structure was different from that in phase 1 with, now, cluster 1 cells increasing (71.61%) and cluster 2 and 3 cells decreasing in abundance (12.26% and 5.04%).

Phase 5 comprised 54 days and was characterized by the pH change of the glycolate feed solution from 3 to 7. This change immediately resulted in increased levels of glyoxylate, propionate, and acetate as well as a clear decrease in biogas productivity. The microbial community responded within only few days with a changed pattern, where the cluster 3 cells increased their abundances dramatically (11.33%). This reactor failure was corrected by a backshift of the feed pH to 3 on day 428.

In phase 6, surprisingly high acid concentrations were still detected for more than 50 days. Especially glyoxylate remained at elevated levels until day 502. Only then the cytometric community structures changed to those patterns observed in phase 4, and the stable biogas productivity and decreased acid concentrations to nearly zero proposed a re-established stable biogas production process.

**Figure 4:**
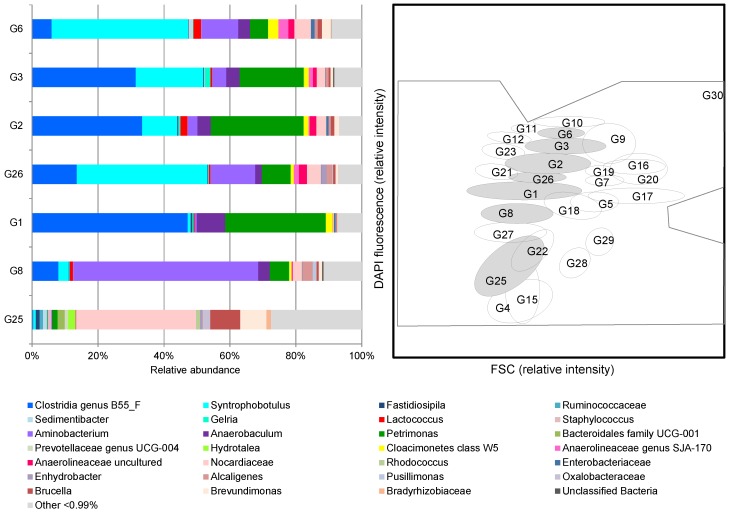
Phylogenetic composition of seven sorted sub-communities (G1–G3, G6, G8, G25, G26) in ascending order based on their position in the cytometric dot plot (DAPI fluorescence intensity). Sub-communities that were sorted are marked in the gate template by a light grey background. Taxonomic affiliation of OTUs is shown on the genus level or on the best achievable taxonomic rank. Only OTUs with a relative abundance of at least 0.99% in at least one sub-community are depicted while the remaining OTUs are summarized to “Other”.

Amplicon sequencing of bacterial 16S rRNA genes and *mcrA* genes was performed exemplarily on sorted cells from gates taken from samples of phase 6 on day 495 (G1, G2, G26: cluster 1 cells) and day 508 (G3, G6: cluster 1 cells, and G8, G25: cluster 2 cells). Theses gates were the most abundant ones during the whole process. As shown in Figure 4, abundant bacterial taxa differed in their proportions in the respective sub-communities.

An OTU assigned to the class Clostridia (genus B55_F) was predominant in sub-communities G1 (47%), G2 (33%), and G3 (31%) and still comprised 14% of G26. The genus *Petrimonas* (phylum Bacteroidetes) was also highly abundant in these three sub-communities with 31%, 28%, and 19%, respectively. In contrast, sub-communities G6 and G26 were dominated by the genus *Syntrophobotulus* (class Clostridia) with 41% and 40%, respectively. Sub-community G8 was with 56% dominated by the genus *Aminobacterium* (phylum Synergistetes). The genus *Syntrophobotulus* was also abundant in sub-communities G2 (11%) and G3 (21%), while *Aminobacterium* with 13% in G26 and 11% in G6 made up substantial parts of specific sub-communities as well. An OTU assigned to the family Nocardiaceae (phylum Actinobacteria) was specifically abundant in sub-community G25 with 36% but comprised only between 1% and 5% in the other sub-communities. Further, abundant OTUs in G25 were the genera *Anaerobaculum* (phylum Synergistetes) with 0–9%, *Candidatus Cloacamonas* (candidate phylum Cloacimonetes; 0–3%), and the alphaproteobacterial genera *Brevundimonas* (8%) and *Brucella* (9%). The detailed OTU table is given in Supplemental Table S8.

Regarding the methanogenic archaea, all sorted sub-communities were dominated by hydrogenotrophic methanogens of the genus *Methanobacterium*, which comprised 90.1% to 99.9% of all *mcrA* OTUs, while some other hydrogenotrophic genera (*Methanoculleus*, *Methanocorpusculum* and *Methanobrevibacter*) were represented by less than 1% in some of the sub-communities (Supplemental Table S9). Only sub-communities G2 and G25 contained substantial percentages (4.9% and 8.9%, respectively) of another *mcrA* sequence type that was only assigned to the phylum Euryarchaeota but could not be identified at any lower taxonomic level. Surprisingly, acetoclastic methanogens were very rare and only represented by the genus *Methanosarcina* with relative abundances between 0% and 0.3%. From these data, it can be concluded that methane was primarily produced via the hydrogenotrophic pathway from hydrogen that was formed as direct or indirect fermentation product of glycolate.

While the amplicon sequencing data gave snapshots of the community composition, the flow cytometric data revealed dynamic variations in community structures that corresponded to changes in the reactor operation. In short, the defined six phases comprised a stable biogas productivity at increasing GLR (phase 1), reactor breakdown due to glycolate overload (phase 2), recovery of the biogas productivity (phase 3), re-stabilization of the biogas productivity (phase 4), accumulation of short-chain carboxylic acids due to increase of the feed pH to 7 (phase 5), and re-stabilization of the biogas productivity due to decreased typical feed pH 3 (phase 6).

### 3.3. Functional Relationships between Biotic and Abiotic Parameters

**Figure 5:**
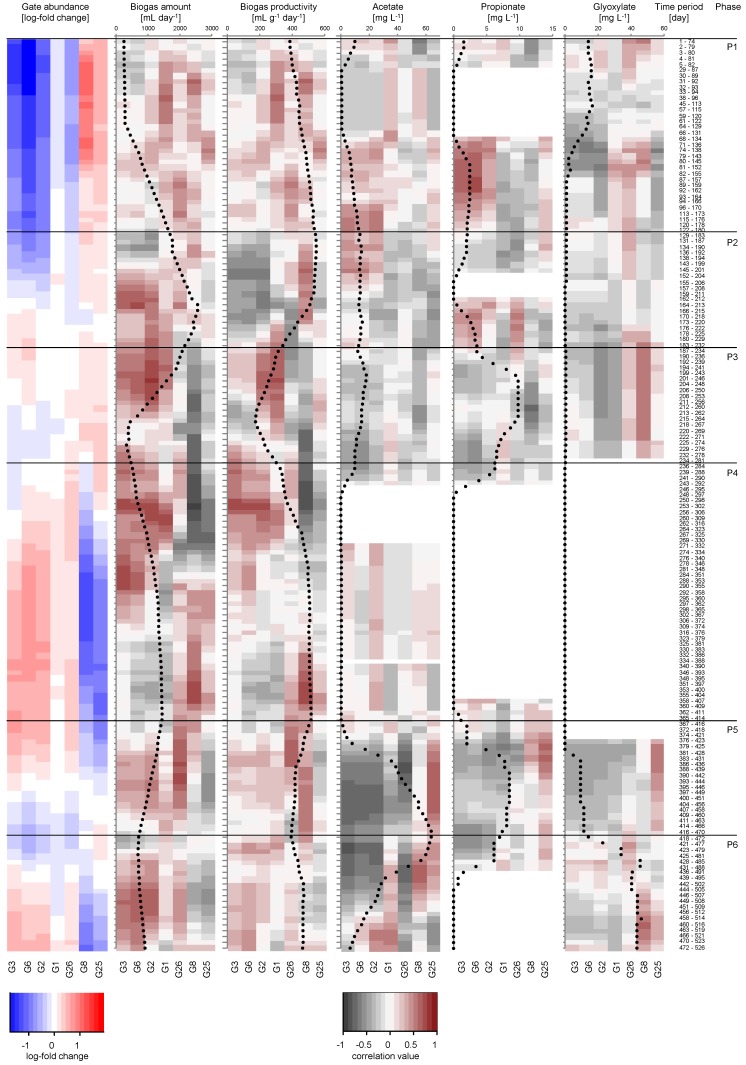
Shown are the abundance (1st heatmap in red and blue) and correlation changes (2nd to 6th heatmap in grey and dark red) of selected seven sub-communities (G1–G3, G6, G8, G25, G26) and five abiotic parameters (biogas productivity, as well as daily biogas amount, acetate, propionate, and glyoxylate production) over time. Six different phases (P1–P6) were identified based on process parameters: P1: days 1–180, P2: days 183–232, P3: days 234–281, P4: days 284–414, P5: days 416–470, and P6: days 472–526 and are marked with lines directly below the respective phases. A moving window correlation analysis with a window size of 20 samples was applied. Correlations were performed using Spearman’s rank order correlation coefficient rho. Color key: average: white (=0); higher values: increasing depth of red color (>0, positive); lower values: increasing depth of grey color (<0, negative). Mean values for all tested parameters are shown as an overlay for each window frame.

To clarify the role of the seven most abundant sub-communities in the formation of biogas and carboxylic acids such as acetate, propionate, and glyoxylate, biotic parameters (cell abundances in sub-communities) and abiotic parameters (acids: mg L^−1^, biogas productivity: mL g^−1^ day^−1^, daily biogas amount: mL day^−1^) in the respective six phases were correlated using the Spearman’s rank order correlation coefficient rho (Figure 5). All sub-communities belonged to clusters 1 and 2. Cluster 3 cells were not sorted due to their low gate cell abundance (Supplemental Table S7).

Only correlations with rho values > 0.4 and *p*-values < 0.05 were further taken into account (all significant correlation values are given in Supplemental Table S10). The produced biogas amount was low in phase 1 (average 697.07 mL/day) and correlated positively with G1 and G26 (cluster 1) cells (peak rho values: G1: 0.64, G26: 0.52). Positive correlations for propionate production were found especially for G3 and G6 cells (peak rho values: G3: 0.63, G6: 0.71), which seem to also be involved in acetate production (peak values: G3: 0.59, G6: 0.46) at the end of phase 1. In contrast, glyoxylate production seems to be related to activities of cells in G8 (peak rho values: G8: 0.64). For glyoxylate production by G8, these findings seem to be valid for the whole process, while propionate formation also correlated positively with cells in G25 but only in phase 5 (peak rho value: G25: 0.61). Additionally, acetate formation shifted to gates G8 and G25 in phases 5 and 6 (peak rho values: G8: 0.66, G25: 0.67).

The daily biogas amount, however, seemed to be mainly related to cells detected in G1–G3 (peak rho values: G1: 0.76, G2: 0.83, G3: 0.72) and G6 (peak rho value: G6: 0.72), which were also the most abundant ones starting from phase 2. This activity was constant until the middle of phase 4. Phase 4 was the period when the process was considered stable due to constant and high biogas amounts (1178.6 mL/day) and stable cytometric community patterns. However, the correlation with the daily biogas amount seemed to shift in the middle of phase 4 mainly to G26 and G8 (peak rho values: G26: 0.47, G8: 0.77). Only the disturbances occurring in phase 5, caused by the pH shift in the glycolate feed to pH 7, seem to cause a reorganization of the function in a way that again G2, G3, G6 and also G26 were the gates that seemed to be responsible for the biogas amount (peak rho values: G2: 0.64, G3:0.61, G6: 0.67, G26: 0.76) until the end of the process. Therefore, cluster 1 cells can probably be attributed to biogas production and partly to acetate and propionate production. Cluster 2 cells showed positive correlations especially with glyoxylate production, but also, to a minor degree, to acetate and propionate production in phases 5 and 6.

## 4. Discussion

Biogas reactors are usually fed with plant biomass such as energy crops, grass silage or landscape conservation material, or organic waste, such as manure, biowaste, agro-industrial residues or wastewater sludge [50]. The process comprises four metabolic steps executed by different microbial consortia, i.e., hydrolysis, acidogenesis, acetogenesis, and methanogenesis [50]. However, anaerobic glycolate metabolization for methane production is a new concept based on basically infinite resources when connected to an algae reactor producing glycolate from CO_2_ and sun light [7]. Such a biogas production process does not need the hydrolysis step and immediately starts with acidogenesis producing several intermediates such as glyoxylate, propionate, and acetate. The products of glycolate fermentation can be either used directly by methanogens (in case of acetate, H_2_, and CO_2_) or further metabolized by syntrophic bacteria (e.g., in case of propionate). The microbial degradation of glycolate usually starts with the oxidation to glyoxylate, which can be further metabolized by three different pathways: (i) The conversion to glycerate, which can be used to form pyruvate and eventually various fermentation products such as acetate, formate, H_2_ and CO_2_ [12]; (ii) the formation of malate by malate synthase and further conversion to pyruvate and subsequent fermentation products as mentioned in (i) [8]; and (iii) the formation of oxaloacetate via the β-hydroxyaspartate pathway [15]. An alternative pathway in Firmicutes is the direct fermentation of glycolate to acetate and succinate without an external electron acceptor as described for a Lachnospiraceae bacterium [19]. Besides glyoxylate and acetate, we detected propionate as intermediate, which is not an intermediate of the aforementioned pathways but can be formed from succinate [51]. Propionate is a critical intermediate in anaerobic digestion as its degradation depends on the syntrophic interaction of propionate-oxidizing bacteria and hydrogenotrophic methanogens that keep the hydrogen partial pressure sufficiently low to enable propionate oxidation to acetate [52].

In the relatively stable phases 4 and 6 of our system, we measured methane contents of 39–45% in the biogas produced from glycolate. Biogas from conventional anaerobic digesters usually has higher methane contents. Substrates such as cattle manure and maize silage produce methane contents up to 55–70% [50]. The differences are caused by the more reduced state (higher C:O ratio) of complex organic substrates. However, complex substrates are prone to cause fluctuations in methane production due to altering composition or the presence of inhibiting components, while the mono-substrate glycolate might have the advantage to always produce stable CH_4_ amounts, which may facilitate process control in future. For complete glycolate conversion to biogas and without considering microbial biomass turnover and acid by-production, the theoretical stoichiometric ratio of CH_4_ to CO_2_ is 3:5 assuming that all electrons of the glycolate would be used for the formation of methane. However, we clearly found biomass turnover as verified by the cytometric pattern analysis. Thus, glycolate was also used for biomass synthesis and subsequently microbial biomass was also converted to methane. The relatively high amounts of organic acids were mainly produced in instable phases of the process and thus represent intermediates that accumulated transiently during instable process periods. We need to state that the 526-day study was performed as a single approach but the 178 biological samples taken in addition with the even higher numbers of abiotic data allowed the verification of trends in community behavior by avoiding indiscriminate sample handling and interpretation.

It was surprising to discover the high diversity of our community on the mono-substrate glycolate, which revealed as much as 29 sub-communities present during the 526 days of cultivation. The structure of the community was evaluated using only the 18 most dominant sub-communities, which were by no means constant in cell abundance and changed in high proportions. Natural communities can be expected to behave very differently when grown on mono-substrates. However, so far there is no experience around on how communities develop under such conditions. Cytometric cluster 1 cells were highly abundant by quota and can be assumed, due to the performed correlation analyses (Figure 5), to be major functional contributors to biogas production but also to acetate and propionate production. In contrast, correlation of cluster 2 cells with abiotic data revealed a tight connection to glyoxylate formation. Cluster 3 cells did not show high correlation values and are therefore assumed to be of lower importance in glycolate fermentation. Therefore, only gates from cell clusters 1 and 2 were used for amplicon sequencing of 16S rRNA and *mcrA* genes revealing a snapshot of the phylogenetic composition of the sub-communities in G1-G3, G6, G8, G25, and G26 (Figure 4). Sub-communities G2, G3, G6, and G26 contained substantial shares of a bacterial OTU assigned to the genus *Syntrophobotulus*, which comprises the syntrophic glycolate-oxidizing species *S. glycolicus* that converts glycolate to H_2_ and CO_2_ in a methanogenic co-culture [8,9,16]. Together with the observed dominance of hydrogenotrophic methanogens, this result suggests that a substantial portion of glycolate was directly converted to methane by a syntrophic consortium of *Syntrophobotulus* and *Methanobacterium*. However, the sub-communities G1–G3 were dominated by the clostridial genus B55_F, the physiological function of which is yet unknown. This genus was detected in substantial proportions in all sub-communities except G25. Based on the known metabolic diversity of the class Clostridia, it can be speculated that it might be a homoacetogen that utilized glycolate directly to form acetate, as described for *Morella* [17,18], a succinate and acetate-producing fermenter as described for a Lachnospiraceae species [19], or a syntrophic acetate-oxidizing bacterium (SAOB) that degrades acetate formed from glycolate by other fermenting bacteria. As almost no acetoclastic methanogens were detected, syntrophic acetate oxidation must be the major acetate sink in the system. The *mcrA* primers we used to detect methanogens were already described in 2002 [37] and thus might have failed to detect more recently described taxa such as Methanomassiliicoccales, Candidatus Methanofastidiosales and Candidatus Verstraetearchaeota. However, these newly described methanogens are probably obligate methylotrophs [53] and thus we did not expect that they play a major role in our system. An abundant genus in sub-communities G1–G3 was *Petrimonas*, members of which have been described as carbohydrate-fermenting bacteria that produce acetate and propionate [54,55]. Being involved in acidogenesis in biogas reactors [55], this genus might be attributed to microbial biomass decay in our system. Another genus that was predominant in G8 but also quite abundant in G6 and G26 was *Aminobacterium*. Species of this genus have been isolated from anaerobic digesters and ferment amino acids to acetate and propionate or oxidize amino acids in syntrophy with *Methanobacterium* that was the prevailing methanogen in our reactor [56,57,58]. A similar physiology has been described for the genus *Anaerobaculum*, which was also present in most sub-communities with abundances of up to 9%. Species of this genus are strictly anaerobic, chemo-organotrophic bacteria fermenting several sugars, peptides, and organics acids to acetate and hydrogen [59,60,61]. Neither glycolate nor one of the metabolites detected in our reactor (glyoxylate, acetate, or propionate) was described to be a substrate of *Aminobacterium* or *Anaerobaculum* species. Thus, it is more likely that these members of the phylum Synergistes were related to microbial biomass decay together with fermenting species of the phylum Bacteroidetes (i.e., mainly *Petrimonas*), and that acetate and propionate accumulated during instable process periods due to biomass turn-over rather than being fermentation products of glycolate. However, propionate could be also formed as fermentation product from succinate provided that our reactor harbored a bacterium fermenting glycolate to succinate and acetate as described for the *Lachnospiraceae* sp. strain 19gly4 [19]. Despite propionate concentrations transiently increased during instable process periods, we did not find any of the classical syntrophic propionate oxidizers that are usually involved in propionate turn-over in biogas reactors digesting complex biomass (e.g., *Syntrophobacter* or *Pelotomaculum*). However, an OTU affiliated to the Cloacimonetes (previously known as candidate division WWE1) was detected in most sub-communities with abundances between 1% and 3%. While the only described species of this phylum (Candidatus *Cloacamonas acidaminovorans*) has been described as syntrophic amino acid degrader based on its genome, it was also discussed as facultative syntrophic propionate oxidizer [62]. However, more detailed analyses such as metagenomics or proteomics would be required to unravel the ecophysiological functions of the several phylotypes in the sub-communities. Nevertheless, the limited taxonomic information we retrieved from MiSeq amplicon sequencing implies that glycolate is most likely converted to methane by syntrophic consortia of *Syntrophobotulus* and *Methanobacterium*, as previously described [8,9,16].

High biogas production is of huge commercial interest; therefore, we started two attempts to increase the biogas productivity. The increase of the glycolate loading rate is one way, and we found indeed higher biogas production with increasing GLR. However, this strategy often fails [63] for high organic loading rates as was also found for our system in phase 2. Acid concentrations increase in a biogas reactor due to the low metabolic capacity of the methanogenic archaea, which are usually present at low cell concentrations and cannot adapt fast enough to new conditions because of their long generation times. In turn, the increasing acid concentrations further inhibit the methane production by archaea [64,65]. Therefore, as the metabolism of methanogens is favored by neutral to slightly alkaline pH values a second strategy was to increase the biogas production by adjusting the pH of the glycolate feed suspension to seven. By this way, forestalling a pH drop in the reactor at prospective higher glycolate loading rates was anticipated. However, although the reactor pH reached values well above 8, biogas productivity decreased (phase 5) and a dramatic increase in acetate and propionate concentrations was observed. Additionally, the CH_4_ content of the biogas increased. The neutralization of the glycolate feed suspension may have led to a shift in the carbonate buffer system due to the higher pH and consequently to lower CO_2_ contents in the biogas. It is also conceivable that the change in the pH glycolate feed led to an increased decay of biomass changing the gas composition in favor of CH_4_. Nevertheless, the overall biogas productivity decreased. While a higher CH_4_ to CO_2_ ratio would be of interest for the industry, a reduction of the biogas productivity by half is obstructive.

## 5. Conclusions

Glycolate is clearly a suitable substrate for several anaerobic microorganisms eventually leading to methane production. The experimental set up and the use of flow cytometry and the connected bioinformatic evaluations disclosed an ingenious and cytometrically diverse ecosystem where mainly the dominant sub-communities jointly contributed to a successful carbon conversion to methane. The high cytometric community structure diversity seems to be an important factor for the continuation of the process. Flow cytometry also reveals vulnerable community states [66], as found in this study for biogas productivity and acid accumulation, which emerged when the glycolate loading rate surpassed 1.8 g L^−1^ day^−1^, a condition that can be easily controlled in a mono-feed operating system. Cell sorting and amplicon sequencing uncovered that the conversion of glycolate to methane was performed mainly by glycolate-fermenting clostridia of the genus *Syntrophobotulus* in syntrophy with hydrogenotrophic methanogens of the genus *Methanobacterium.*

## Supplementary Materials

The following are available online at http://www.mdpi.com/2076-2607/6/4/103/s1, Figure S1: Design of the glycolate-fed biogas reactor (5 L) with a working volume of 3.5 L, Table S2: Media and buffer compositions of the feed solution, Table S3: Productivity, process products, abiotic and biotic parameters measured over 526 days, Table S4: Sorted sampling days and gates, Table S5: DNA extraction and library preparation for Illumina MiSeq sequencing, Table S6: Euclidian distances of dominant gates shown in Figure 3. Table S7: Cluster abundances per phase, Table S8: OTU abundances: Summary 16S rRNA gene sequence analysis, Table S9: OTU abundances: Summary *mcrA* gene sequence analysis, Table S10: Significant correlation values.
