# Supplementary material for: Long-Term Biogas Production from Glycolate by Diverse and Highly Dynamic Communities
Source: Microorganisms. 2018 Oct 4;6(4):103. doi: 10.3390/microorganisms6040103 (PMC6313629; doi:10.3390/microorganisms6040103)
Supplement: Supplementary file 1 [file microorganisms-06-00103-s001.zip › microorganisms-356095-suppl-proofreading done/S10.pdf]

Table S10. Significant correlation values. Correlation values (Spearmans rho) corresponding to the correlation maps in Figure 5 with only the significant (*P*-value after Benjamini-Hochberg correction <0.05) correlations shown for each parameter and time point. A moving window approach was used for the analysis with 20 time points as the window size. Phases (P1: day 1-180, P2: day 183-232, P3: day 234-281, P4: day 284-414, P5: day 416-470, P6: day 472-526) can be differentiated by the alternating grey/white background coloring.

Time range	Biogas amount							Biogas productivity							Acetate							Propionate							Glyoxylate						
	G3	G6	G2	G1	G26	G8	G25	G3	G6	G2	G1	G26	G8	G25	G3	G6	G2	G1	G26	G8	G25	G3	G6	G2	G1	G26	G8	G25	G3	G6	G2	G1	G26	G8	G25
1 - 74																				-0.64							-0.60						0.45	0.50	
2 - 79																				-0.60							-0.60								
3 - 80																				-0.54							-0.54								
4 - 81																											-0.46								
5 - 82																																			
29 - 87				0.48							0.48																								
30 - 89						0.49							0.49																						
31 - 92				0.49		0.60					0.49		0.60																						
32 - 93				0.53		0.60					0.53		0.60																						
33 - 94				0.51		0.58					0.51		0.58																						
38 - 96				0.64							0.64																								
45 - 113				0.53							0.53																								
57 - 115				0.50							0.50																								
59 - 120				0.54							0.54																								
61 - 122				0.52							0.52																								
64 - 129																																			
66 - 131																																			
68 - 134				0.47																									-0.53	-0.56					
71 - 136							0.47																0.50						-0.55	-0.65	-0.57				
74 - 138							0.59							0.52								0.52	0.58	0.50					-0.53	-0.61	-0.62				-0.61
79 - 143							0.49															0.57	0.63							-0.54	-0.51				
80 - 145																						0.63	0.68						-0.56	-0.56	-0.55			0.53	
81 - 152																						0.62	0.69						-0.51	-0.51	-0.67				-0.50

82 - 155																						0.62	0.69								-0.57				
87 - 157																						0.61	0.71												
89 - 159					0.52							0.52										0.61	0.71												
92 - 162					0.47							0.48										0.62	0.71												
93 - 164					0.49							0.49										0.59	0.55												
94 - 166																						0.54													
96 - 170					0.46																	0.50													
113 - 173					0.51							0.47			0.50		0.52																		
115 - 176															0.51	0.46	0.54																		
120 - 178															0.52		0.54																		
122 - 180															0.59		0.55																		
129 - 183			-0.53																							-0.45									
131 - 187			-0.46																							-0.54									
134 - 190		-0.56	-0.55												0.49											-0.56									
136 - 192									-0.52	-0.48															-0.52	-0.50									
138 - 194								-0.53	-0.56	-0.55			0.49												-0.47										
143 - 199								-0.57	-0.57	-0.60			0.55		0.47	0.48																			
145 - 201								-0.61	-0.60	-0.64			0.62		0.51	0.52				-0.45															
152 - 204								-0.66	-0.66	-0.69			0.50							-0.45															
155 - 206										-0.53			0.51							-0.45															
157 - 208	0.55	0.55	0.47					-0.46	-0.47	-0.57			0.49								-0.44														
159 - 211	0.62	0.62	0.53					-0.49	-0.51	-0.62			0.54								-0.51														
162 - 212	0.67	0.69	0.61						-0.47	-0.61			0.49								-0.48														
164 - 213	0.69	0.71	0.59		0.51			-0.46	-0.49	-0.65	-0.52	-0.46	0.47	-0.46																					
166 - 215	0.47		0.59					-0.48	-0.54	-0.54		-0.52	0.51								-0.50					0.48									
170 - 218			0.50					-0.50	-0.58	-0.50		-0.51	0.54					-0.46			-0.58	0.47	0.53			0.51									
173 - 220			0.49						-0.55														0.50			0.48									
176 - 222			0.54									-0.51									-0.49					0.51					-0.52				
178 - 225			0.56									-0.58																							

180 - 229			0.60	0.47							0.47	-0.56									-0.48				-0.48										
183 - 232			0.66	0.49							0.59	-0.56								-0.45	-0.47										-0.52	-0.46		0.52	
187 - 234	0.49	0.48	0.67	0.53							0.62	-0.56								-0.44											-0.52	-0.46		0.52	
190 - 236	0.52	0.51	0.70	0.57						0.51	0.66	-0.53								-0.46											-0.52	-0.46		0.52	
192 - 239	0.51	0.51	0.72	0.57		-0.47				0.57	0.64	-0.46																			-0.52	-0.46		0.52	
194 - 241	0.59	0.57	0.77	0.60		-0.51				0.60	0.67																				-0.52	-0.46		0.52	
199 - 243	0.61	0.60	0.79	0.54		-0.50				0.63	0.69	-0.45																			-0.52	-0.46		0.52	
201 - 246	0.59	0.63	0.83	0.51		-0.56				0.66	0.73																				-0.52	-0.46		0.52	
204 - 248	0.51	0.63	0.79	0.53		-0.55				0.65	0.68																-0.52				-0.52			0.52	
206 - 250	0.47	0.60	0.73			-0.63				0.57	0.59																-0.51				-0.52			0.52	
208 - 253		0.56	0.70			-0.76				0.55	0.63																-0.51				-0.52			0.52	
211 - 256		0.48	0.65			-0.76				0.45	0.60																-0.58				-0.52			0.52	
212 - 260			0.55			-0.72					0.58									-0.50							-0.72				-0.52			0.52	
213 - 262						-0.69					0.51									-0.57							-0.67				-0.52			0.52	
215 - 264						-0.66					0.46		-0.48							-0.55							-0.61				-0.46			0.52	
218 - 267					-0.54	-0.59							-0.59							-0.49							-0.54				-0.46	-0.46		0.52	
220 - 269					-0.55	-0.59						-0.45	-0.74			-0.47															-0.46	-0.46		0.52	
222 - 271						-0.74							-0.74			-0.48							-0.47								-0.46	-0.46		0.52	
225 - 274						-0.74							-0.71		-0.47	-0.52							-0.52												
229 - 276						-0.74							-0.70		-0.47	-0.52							-0.51												
232 - 278	0.53					-0.74		0.46					-0.70		-0.47	-0.55						-0.48	-0.51												
234 - 281	0.59					-0.73		0.53					-0.69		-0.49	-0.54						-0.49	-0.54												
236 - 284	0.59	0.45				-0.77		0.51					-0.73		-0.45	-0.50							-0.49												
239 - 288	0.62	0.54			0.46	-0.80		0.63	0.49				-0.75																						
241 - 290	0.53					-0.81		0.58					-0.77																						
243 - 292	0.47					-0.87		0.53					-0.83																						
246 - 295						-0.87		0.53					-0.84																						
248 - 297		0.47	0.52			-0.88	-0.47	0.53	0.47	0.50			-0.83																						
250 - 298	0.70	0.65	0.67			-0.89	-0.61	0.76	0.65	0.64			-0.83	-0.51																					

253 - 302	0.72	0.72	0.78	0.56		-0.90	-0.64	0.75	0.70	0.71			-0.81	-0.51																					
256 - 306	0.65	0.70	0.78	0.69		-0.87	-0.63	0.66	0.66	0.68	0.53		-0.74																						
260 - 309			0.74	0.72		-0.83	-0.64			0.63	0.55		-0.68																						
262 - 316		0.53	0.67	0.76		-0.80	-0.64		0.50	0.56	0.57		-0.62																						
264 - 323	0.49	0.53	0.58	0.62		-0.78	-0.69		0.50	0.53	0.50		-0.56																						
267 - 325	0.49	0.49	0.55	0.55		-0.75	-0.71						-0.49																						
269 - 330	0.54	0.51	0.53		-0.57	-0.73	-0.72																												
271 - 332	0.58		0.57		-0.68	-0.62	-0.65																												
274 - 334	0.58	0.49			-0.56	-0.49	-0.53																												
276 - 340	0.61																																		
278 - 346	0.67	0.50																																	
281 - 348	0.70	0.56																																	
284 - 351	0.72	0.69							0.48																										
288 - 353	0.72	0.69																																	
290 - 355	0.71	0.69		-0.48																															
292 - 358	0.55	0.50		-0.50																															
295 - 360				-0.48																															
297 - 362				-0.47																															
298 - 365																																			
302 - 367																																			
306 - 372																																			
309 - 374																																			
316 - 376																																			
323 - 379						0.46																													
325 - 381						0.57																													
330 - 383						0.54																													
332 - 386						0.53							0.53																						
334 - 388						0.54							0.54																						
340 - 390						0.56							0.56																						

346 - 393						0.62							0.62																						
348 - 395						0.67							0.67																						
351 - 397						0.77							0.77																						
353 - 400						0.76							0.76																						
355 - 404						0.76							0.76																						
358 - 407						0.73							0.73																						
360 - 409						0.55							0.55																						
362 - 411																																			
365 - 414					0.47							0.47																							
367 - 416																																			
372 - 418					0.56							0.56																							
374 - 421					0.57							0.57													-0.50										
376 - 423					0.61							0.61							-0.58					-0.49	-0.56			0.55							
379 - 425					0.64							0.64							-0.70					-0.57	-0.60	-0.50		0.60	-0.47	-0.48	-0.47		-0.47		0.53
381 - 428					0.65							0.65			-0.46	-0.49	-0.54		-0.73		0.51			-0.53	-0.59			0.54	-0.47	-0.48	-0.47				0.53
383 - 431					0.70		-0.46					0.65			-0.57	-0.60	-0.58		-0.79		0.56	-0.49	-0.46	-0.59		-0.53		0.61	-0.47	-0.48	-0.47				0.53
386 - 436					0.76		-0.49					0.64			-0.64	-0.63	-0.61		-0.83		0.60	-0.48		-0.54				0.57	-0.47	-0.48	-0.47				0.53
388 - 439					0.69		-0.47						0.52		-0.66	-0.64	-0.65		-0.81		0.63			-0.49	-0.46			0.54	-0.47	-0.48	-0.47				0.53
390 - 442			0.47		0.70		-0.53						0.53		-0.67	-0.66	-0.66		-0.78		0.61								-0.47	-0.48					0.47
393 - 444			0.51		0.70		-0.56						0.50		-0.74	-0.70	-0.69	-0.48	-0.74		0.63									-0.47					
395 - 446	0.46		0.54		0.64		-0.60						0.52		-0.78	-0.73	-0.72	-0.46	-0.70		0.66									-0.47					
397 - 449			0.49		0.58		-0.55						0.54		-0.78	-0.73	-0.72	-0.48	-0.68		0.67														
400 - 451			0.46		0.55		-0.52						0.57		-0.77	-0.75	-0.73	-0.53	-0.61		0.66														
404 - 456					0.51		-0.50						0.54		-0.78	-0.74	-0.73	-0.54	-0.55		0.61														
407 - 458													0.57		-0.80	-0.75	-0.72	-0.51	-0.52		0.60														
409 - 460													0.51		-0.78	-0.72	-0.69	-0.49	-0.45		0.57														
411 - 463												-0.45	0.50		-0.75	-0.69	-0.66	-0.44			0.47														
414 - 466													0.55		-0.66	-0.56	-0.57					-0.56	-0.55	-0.49											
416 - 470													0.49		-0.64	-0.55	-0.58					-0.56	-0.55	-0.49											

418 - 472													0.49		-0.64	-0.56	-0.59					-0.56	-0.55	-0.49											
421 - 477															-0.83	-0.74	-0.79		-0.48			-0.56	-0.55	-0.49									0.45		
423 - 479															-0.81	-0.72	-0.78		-0.49		0.50	-0.45	-0.44												
425 - 481															-0.79	-0.70	-0.77		-0.63		0.47														
428 - 485															-0.73	-0.63	-0.71		-0.62	0.51															
431 - 488															-0.70	-0.61	-0.68		-0.71	0.65															
436 - 491															-0.72	-0.64	-0.68		-0.71	0.66															
439 - 495															-0.69	-0.59	-0.62		-0.71	0.62															
442 - 502	0.49	0.58	0.56				-0.50								-0.63	-0.52	-0.56		-0.69	0.55															
444 - 505	0.54	0.63	0.62				-0.54								-0.56	-0.45	-0.50		-0.62	0.49															
446 - 507	0.59	0.66	0.64				-0.54								-0.49				-0.55																
449 - 508	0.61	0.67	0.64				-0.51												-0.60																
451 - 509	0.59	0.66	0.64				-0.49												-0.57																
456 - 512	0.59	0.65	0.61																-0.50																
458 - 514	0.50	0.60	0.55																		-0.46													0.56	
460 - 516	0.51	0.58																0.47			-0.57													0.64	
463 - 519	0.47	0.59															0.54	0.58			-0.59													0.56	
466 - 521		0.56															0.51	0.55			-0.57													0.55	
470 - 523		0.52															0.49	0.50	-0.54		-0.53													0.47	
472 - 526																		0.47	-0.56		-0.50														

References:
Benjamini, Y.; Hochberg Y. Controlling the false discovery rate: A practical and powerful approach to multiple testing. *J. R. Stat. Soc. Ser. B Stat. Methodol.* **1995**, *57*, 289–300.
