# Supplementary material for: Long-Term Biogas Production from Glycolate by Diverse and Highly Dynamic Communities
Source: Microorganisms. 2018 Oct 4;6(4):103. doi: 10.3390/microorganisms6040103 (PMC6313629; doi:10.3390/microorganisms6040103)
Supplement: Supplementary file 1 [file microorganisms-06-00103-s001.zip › microorganisms-356095-suppl-proofreading done/S2.pdf]

Table S2. Media and buffer composition. Media composition of the feed solution (pH was adjusted to 7.2 with NaOH) and composition of the phosphate-buffered saline (PBS, pH adjusted to 7.2 with either NaH_2PO_4 or Na_2HPO_4).

Component	Concentration	
Salts		
KH_2PO_4	0.2	$g\ L^{-1}$
NH_4Cl	0.25	$g\ L^{-1}$
NaCl	1	$g\ L^{-1}$
$MgCl_2 \cdot 6H_2O$	0.4	$g\ L^{-1}$
KCl	0.5	$g\ L^{-1}$
$CaCl_2\ 2H_2O$	0.15	$g\ L^{-1}$
Cysteine-HCl	0.27	$g\ L^{-1}$
Trace elements		
$FeCl_2 \cdot 4H_2O$	3	$\mu g\ L^{-1}$
$ZnCl_2$	140	$\mu g\ L^{-1}$
$MnCl_2\ 4H_2O$	0.2	$mg\ L^{-1}$
H_3BO_3	12	$\mu g\ L^{-1}$
$CoCl_2\ 6H_2O$	0.38	$mg\ L^{-1}$
$CuCl_2 \cdot 2H_2O$	4	$\mu g\ L^{-1}$
$NiCl_2\ 6H_2O$	48	$\mu g\ L^{-1}$
$Na_2MoO_4\ 2H_2O$	72	$\mu g\ L^{-1}$
Vitamins		
Vitamin B12	50	$\mu g\ L^{-1}$
p-Aminobenzoic acid	40	$\mu g\ L^{-1}$
D(+)-Biotin	10	$\mu g\ L^{-1}$
Nicotinic acid	100	$\mu g\ L^{-1}$
Calcium pantothenate	50	$\mu g\ L^{-1}$
Pyridoxine hydrochloride	150	$\mu g\ L^{-1}$
Thiamine-HCl $2H_2O$	100	$\mu g\ L^{-1}$
Folic acid	15	$\mu g\ L^{-1}$
Lipoic acid	5	$\mu g\ L^{-1}$

Component	Concentration	
NaH_2PO_4	0.22	$g\ L^{-1}$
Na_2HPO_4	0.85	$g\ L^{-1}$
NaCl	8.77	$g\ L^{-1}$
