# Supplementary material for: Long-Term Biogas Production from Glycolate by Diverse and Highly Dynamic Communities
Source: Microorganisms. 2018 Oct 4;6(4):103. doi: 10.3390/microorganisms6040103 (PMC6313629; doi:10.3390/microorganisms6040103)
Supplement: Supplementary file 1 [file microorganisms-06-00103-s001.zip › microorganisms-356095-suppl-proofreading done/S3_SK.docx]

**Table S3.**Productivity, process products, abiotic (a) and biotic (b) parameters measured over 526 days.

Phases (P1: day 1-180, P2: day 183-232, P3: day 234-281, P4: day 284-414, P5: day 416-470, P6: day 472-526) can be differentiated by the alternating grey/white background coloring.

**Table S3a.** Productivity, process products and abiotic parameters.

A – Acetate, P – Propionate, G – Glyoxylate, GLR – Glycolate Loading Rate, HRT – Hydraulic Retention Time, RT – Room Temperature, VOA/TIC - Volatile Organic Acids / Total Inorganic Carbon. RT and air pressure values served for normalization of the biogas amount to standard conditions.

|  | Productivity | Products | | | | | | Abiotic parameters | | | | | Values for biogas amount correction | |
| --- | --- | --- | --- | --- | --- | --- | --- | --- | --- | --- | --- | --- | --- | --- |
| Day | Biogas productivity | Biogas  amount | CH_4_ | CO_2_ | A | P | G | GLR | HRT | pH | VOA/TIC | pH | RT | Air pressure |
|  | [mL g^-1^ d^-1^] | [mL d^-1^] | [%] | [%] | [mg L^-1^] | [mg L^-1^] | [mg L^-1^] | [g L^-1^ d^-1^] | [d] |  |  | feed | [°C] | [hPa] |
| 1 | 361.8 | 226.1 | 44.3 | 53.9 | 14.4 | 0 | 0 | 0.179 | 416.7 | 7.4 | 0.1 | 3 | 19.8 | 1003.4 |
| 2 | 372.1 | 232.6 | 44.9 | 53.1 | 47.4 | 8 | 0 | 0.179 | 416.7 | 7.5 | 0.2 | 3 | 19.9 | 1003.4 |
| 3 | 351.4 | 219.6 | 44.9 | 53 | 41.7 | 6.5 | 0 | 0.179 | 416.7 | 7.5 | 0.1 | 3 | 20.1 | 1009.6 |
| 4 | 403.1 | 251.9 | 45.1 | 53 | 42.9 | 6.6 | 0 | 0.179 | 416.7 | 7.4 | 0.1 | 3 | 20.3 | 998 |
| 5 | 397.9 | 248.7 | 44.8 | 53.1 | 50.8 | 9.6 | 0 | 0.179 | 416.7 | 7.7 | 0.1 | 3 | 20.1 | 996.5 |
| 29 | 286.7 | 179.2 | 44.4 | 53 | 0 | 0 | 0 | 0.179 | 416.7 | 7.5 | 0.1 | 3 | 23.5 | 1017 |
| 30 | 368.6 | 230.4 | 43.6 | 53.7 | 0 | 0 | 36.1 | 0.179 | 416.7 | 7.9 | 0.2 | 3 | 21.6 | 1017.2 |
| 31 | 389.1 | 243.2 | 43.8 | 53.1 | 0 | 0 | 0 | 0.179 | 416.7 | 7.6 | 0.1 | 3 | 19.8 | 1018.8 |
| 32 |  |  | 44 | 53.7 | 0 | 0 | 0 | 0.179 | 416.7 | 7.5 | 0.2 | 3 | 23.2 | 1008.2 |
| 33 | 317.1 | 198.2 | 43.9 | 53.2 | 0 | 0 | 0 | 0.179 | 416.7 | 7.5 | 0.1 | 3 | 26.5 | 1020.9 |
| 38 | 399.4 | 249.6 | 41.1 | 52 | 0 | 0 | 0 | 0.179 | 416.7 | 7.6 | 0.2 | 3 | 23.8 | 979.1 |
| 45 | 450.6 | 281.6 |  |  | 0 | 0 | 30.2 | 0.179 | 416.7 | 7.5 | 0.1 | 3 | 20.9 | 1007 |
| 57 | 373.8 | 233.6 | 44.6 | 53.1 | 0 | 0 | 28.9 | 0.179 | 416.7 | 7.4 | 0.1 | 3 | 27 | 1006.8 |
| 59 | 419.8 | 262.4 | 43.5 | 54.5 | 0 | 0 | 0 | 0.179 | 416.7 | 7.4 | 0.1 | 3 |  |  |
| 61 | 358.4 | 224 | 43.2 | 55 | 0 | 0 | 17.8 | 0.179 | 416.7 | 7.5 | 0 | 3 |  |  |
| 64 | 409.6 | 256 | 43.6 | 53 | 0 | 0 | 36.4 | 0.179 | 416.7 | 7.8 | 0 | 3 | 27.3 | 1002 |
| 66 | 425 | 265.6 | 43.6 | 53.6 | 0 | 0 | 42.7 | 0.179 | 416.7 | 7.5 | 0.1 | 3 | 27 | 1000.6 |
| 68 | 440.3 | 275.2 | 43.1 | 54.2 | 0 | 0 | 35.1 | 0.179 | 416.7 | 7.7 | 0.1 | 3 | 23.8 | 1008.6 |
| 71 | 404.5 | 252.8 | 43.5 | 54.2 | 0 | 0 | 23 | 0.179 | 416.7 | 7.5 | 0.1 | 3 | 25 | 1014.7 |
| 74 |  |  |  |  | 0 | 0 | 32 | 0.179 | 416.7 | 7.7 | 0.1 | 3 | 26.5 | 1003.1 |
| 79 |  |  | 43.2 | 54.9 | 0 | 0 | 0 | 0.179 | 416.7 | 7.5 | 0.1 | 3 | 25.3 | 1015.5 |
| 80 | 460.8 | 288 |  |  | 0 | 0 | 0 | 0.179 | 416.7 | 7.6 | 0.2 | 3 | 25.6 | 1012.7 |
| 81 | 430.1 | 268.8 |  |  | 10.3 | 0 | 23.4 | 0.179 | 416.7 | 7.8 | 0.2 | 3 | 23.8 | 1002 |
| 82 | 455.7 | 284.8 |  |  | 0 | 0 | 12.7 | 0.179 | 416.7 | 7.6 | 0.1 | 3 | 23.3 | 997.2 |
| 87 | 522.2 | 326.4 |  |  | 0 | 0 | 0 | 0.179 | 416.7 | 7.3 | 0.2 | 3 | 23.9 | 1073.6 |
| 89 | 460.8 | 288 |  |  | 0 | 0 | 0 | 0.179 | 416.7 | 7.7 | 0.1 | 3 | 22.2 | 996 |
| 92 | 522.2 | 326.4 |  |  | 0 | 0 | 0 | 0.179 | 416.7 | 7.7 | 0.1 | 3 | 23.3 | 993.2 |
| 93 | 409.6 | 256 |  |  |  |  |  | 0.179 | 416.7 |  |  | 3 | 21.7 | 985.4 |
| 94 | 425 | 265.6 |  |  | 0 | 0 | 0 | 0.179 | 416.7 | 7.3 | 0.1 | 3 | 22.9 | 983 |
| 97 | 522.2 | 326.4 |  |  |  |  |  | 0.179 | 416.7 |  |  | 3 | 22.2 | 990 |
| 113 | 522.2 | 326.4 |  |  |  |  |  | 0.179 | 416.7 | 7.1 | 0.2 | 3 | 27.7 | 1004 |
| 115 | 384 | 240 |  |  | 0 | 0 | 0 | 0.179 | 416.7 | 7.2 | 0.2 | 3 | 28.2 | 998.3 |
| 120 | 404.5 | 252.8 |  |  | 0 | 0 | 0 | 0.179 | 416.7 | 7.5 | 0.1 | 3 | 24.3 | 1003.2 |
| 122 | 440.3 | 275.2 |  |  |  |  |  | 0.179 | 416.7 | 7.7 | 0.1 | 3 | 26.2 | 1001.2 |
| 129 | 540.9 | 1760 |  |  | 0 | 0 | 0 | 0.930 | 120.5 | 7 | 0.3 | 3 | 24.3 | 1002 |
| 131 | 515.1 | 1676 | 37.9 | 58.2 | 17.5 | 0 | 0 | 0.930 | 120.5 | 7.1 | 0.1 | 3 | 24.2 | 1001 |
| 134 | 544.9 | 1772.8 | 38.7 | 60.3 | 17.5 | 7.2 | 0 | 0.930 | 120.5 | 7.7 | 0.1 | 3 | 28.8 | 988.3 |
| 136 | 573.4 | 1865.6 | 38.9 | 60.2 | 22.8 | 5.9 | 0 | 0.930 | 120.5 | 7.4 | 0.2 | 3 | 30.2 | 1006 |
| 138 | 574.4 | 1868.8 | 38.2 | 59.5 | 25.9 | 10.2 | 0 | 0.930 | 120.5 | 7 | 0.2 | 3 | 27 | 1012.7 |
| 143 | 544.9 | 1772.8 |  |  | 9.4 | 7 | 0 | 0.930 | 120.5 | 7.4 | 0.2 | 3 | 23.1 | 1003 |
| 145 | 544.9 | 1772.8 |  |  | 7.8 | 8.8 | 0 | 0.930 | 120.5 | 7.1 | 0.2 | 3 | 22.5 | 998.8 |
| 152 | 550.8 | 1792 | 37.4 | 57.7 | 11.8 | 0 | 15.7 | 0.930 | 120.5 | 7.7 | 0.2 | 3 | 26.5 | 994 |
| 155 | 570.9 | 1857.6 | 37.2 | 58.6 | 6.2 | 0 | 0 | 0.930 | 120.5 | 7.4 | 0.1 | 3 | 24.4 | 991.2 |
| 157 | 550.8 | 1792 | 37.4 | 55.2 | 12 | 0 | 0 | 0.930 | 120.5 | 7.4 | 0.1 | 3 | 24.1 | 997.4 |
| 159 | 575.4 | 1872 |  |  | 6.6 | 0 | 0 | 0.930 | 120.5 | 7.1 | 0.2 | 3 | 24.6 | 1005 |
| 162 | 542 | 1763.5 | 39.5 | 59.4 | 7 | 0 | 0 | 0.930 | 120.5 | 7 | 0.2 | 3 | 29 | 1000.9 |
| 164 | 546.8 | 1779.2 | 36.4 | 55.3 | 5.9 | 0 | 0 | 0.930 | 120.5 | 7.1 | 0.2 | 3 | 25.2 | 1001 |
| 166 | 560.9 | 1772.8 | 39.6 | 58.9 | 12.5 | 0 | 0 | 0.903 | 124 | 7.4 | 0.2 | 3 | 26.3 | 1004 |
| 170 | 573 | 1811.2 | 39.4 | 59.6 | 23.6 | 0 | 0 | 0.903 | 124 |  |  | 3 | 26.3 | 1005.9 |
| 173 | 518.4 | 1638.4 | 42 | 57.2 | 19.7 | 0 | 0 | 0.903 | 124 |  |  | 3 | 30.8 | 997.1 |
| 176 | 503.2 | 1590.4 | 40.2 | 58.6 | 13.9 | 0 | 1.5 | 0.903 | 124 | 6.9 | 0.2 | 3 | 27.6 | 1006.8 |
| 178 |  |  |  |  | 15.7 | 0 | 0 | 0.903 | 124 | 6.8 | 0.3 | 3 | 28.4 | 1002 |
| 180 | 543.7 | 1718.4 |  |  | 18.5 | 0 | 0 | 0.903 | 124 | 7.1 | 0.2 | 3 | 29.4 | 998 |
| 183 | 499.1 | 1577.6 | 39.3 | 59.9 | 0 | 0 | 0 | 0.903 | 124 | 7.1 | 0.2 | 3 | 26 | 999.2 |
| 187 | 574.1 | 1814.4 | 39.8 | 59.6 | 29.2 | 0 | 0 | 0.903 | 124 | 7.4 | 0.2 | 3 | 22.6 | 995.6 |
| 190 | 510.3 | 1612.8 |  |  | 22.3 | 0 | 0 | 0.903 | 124 | 7 | 0.2 | 3 | 24.1 | 1008.6 |
| 192 | 511.3 | 3232 | 38.2 | 59.2 | 0 | 0 | 0 | 1.806 | 124 |  |  | 3 | 23.9 | 1002 |
| 194 | 518.4 | 3276.8 | 38 | 59.8 | 24.7 | 0 | 0 | 1.806 | 124 | 7.3 | 0.2 | 3 | 24.1 | 1001 |
| 199 | 530 | 3350.4 | 37.3 | 59.7 | 15.9 | 0 | 0 | 1.806 | 124 | 6.8 | 0.3 | 3 | 31.4 | 1001 |
| 201 | 505.7 | 3196.8 | 37.8 | 59.3 | 22 | 0 | 0 | 1.806 | 124 | 8.3 | 0.3 | 3 | 33 | 1001.2 |
| 204 | 478.4 | 3024 |  |  | 0 | 0 | 0 | 1.806 | 124 | 6.9 | 0.3 | 3 | 26 | 1003.1 |
| 206 | 575 | 3635 |  |  | 12.1 | 0 | 0 | 1.806 | 124 | 7.2 | 0.2 | 3 | 28.2 | 1004.7 |
| 208 | 502.2 | 3174.4 |  |  | 11.4 | 0 | 0 | 1.806 | 124 | 6.9 | 0.3 | 3 | 28 | 1003.1 |
| 211 | 262 | 3312 | 38.2 | 59.4 | 0 | 0 | 0 | 3.612 | 124 | 7.1 | 0.4 | 3 | 26.8 | 1008 |
| 212 | 409 | 5171 | 33.9 | 61.1 | 0 | 9.9 | 0 | 3.612 | 124 | 6.1 | 1.7 | 3 | 26.5 | 1003 |
| 213 | 144 | 1820.8 | 37.9 | 58.6 | 16.3 | 13.5 | 0 | 3.612 | 124 | 6.9 | 0.6 | 3 | 25.9 | 1002 |
| 215 | 53.4 | 675.2 | 41.2 | 55.8 | 24.6 | 17.8 | 0 | 3.612 | 124 | 6.9 | 0.3 | 3 | 24.1 | 1010.3 |
| 218 | 25.3 | 320 | 42.9 | 52 | 33 | 11.3 | 0 | 3.612 | 124 | 6.8 | 0.2 | 3 | 25.6 | 1004.1 |
| 220 | 172.4 | 2179.2 | 36.1 | 62.4 | 0 | 6.6 | 0 | 3.612 | 124 | 3.6 |  | 3 | 27 | 1002.5 |
| 222 | 0 | 0 |  |  | 21.8 | 5.9 | 3.9 | 3.612 | 124 | 3.3 |  | 3 | 26.9 | 1009.3 |
| 225 | 0 | 0 | 39.5 | 55.5 | 0 | 0 | 0 | 3.612 | 124 |  |  | 3 | 34 | 1003.2 |
| 229 | 0 | 0 |  |  | 0 | 5.6 | 0 | 3.612 | 124 |  |  | 3 | 25.5 | 994 |
| 232 | 0 | 0 |  |  | 4 | 0 | 5.3 | 3.612 | 124 | 3.3 |  | 3 | 27.4 | 1006.2 |
| 234 | 184.8 | 220.8 |  |  | 35.8 | 16.2 | 0 | 0.341 | 470.4 |  |  | 3 | 31 | 1006.4 |
| 236 | 436.5 | 521.6 |  |  | 65.6 | 32.5 | 0 | 0.341 | 470.4 | 7.3 | 0.5 | 3 | 27.2 | 1002.6 |
| 239 | 364.2 | 435.2 |  |  | 38.9 | 40.2 | 0 | 0.341 | 470.4 | 7 |  | 3 | 30.3 | 101 |
| 241 | 203.5 | 243.2 |  |  | 33.4 | 28.1 | 0 | 0.341 | 470.4 | 7.8 | 0.2 | 3 | 23.7 | 1006 |
| 243 | 238.4 | 284.8 |  |  | 19.4 | 8 | 0 | 0.341 | 470.4 |  |  | 3 | 22.9 | 1008.7 |
| 246 | 291.9 | 348.8 |  |  | 0 | 0 | 0 | 0.341 | 470.4 |  |  | 3 | 29.6 | 1002 |
| 248 | 211.6 | 252.8 |  |  | 0 | 0 | 0 | 0.341 | 470.4 | 7.2 | 0.2 | 3 | 29.9 | 999 |
| 250 | 174.1 | 208 |  |  | 0 | 0 | 0 | 0.341 | 470.4 |  |  | 3 | 23.3 | 1002 |
| 253 | 219.6 | 262.4 |  |  | 0 | 0 | 0 | 0.341 | 470.4 | 7.3 | 0.1 | 3 | 24.6 | 1004.1 |
| 256 | 238.4 | 284.8 |  |  | 0 | 0 | 0 | 0.341 | 470.4 | 7.2 | 0.1 | 3 | 24.5 | 1003 |
| 260 | 134.6 | 214.4 |  |  | 0 | 0 | 0 | 0.455 | 352.8 | 7.1 | 0.1 | 3 | 24.1 | 1005 |
| 262 | 379.6 | 604.8 |  |  | 0 | 0 | 0 | 0.455 | 352.8 | 7.2 | 0.2 | 3 | 24.9 | 989 |
| 264 | 385.7 | 614.4 |  |  | 0 | 0 | 0 | 0.455 | 352.8 | 7 | 0.1 | 3 | 25 | 995.3 |
| 267 | 387.7 | 617.6 |  |  | 0 | 0 | 0 | 0.455 | 352.8 | 7.2 | 0.1 | 3 | 24.9 | 1008.8 |
| 269 | 375.6 | 598.4 |  |  | 0 | 0 | 0 | 0.455 | 352.8 | 6.9 | 0.1 | 3 | 24.5 | 1002.1 |
| 271 | 380.8 | 758.4 |  |  | 0 | 0 | 0 | 0.569 | 282.3 | 6.9 | 0.1 | 3 | 24.6 | 992.6 |
| 274 | 380.8 | 758.4 |  |  | 0 | 0 | 0 | 0.569 | 282.3 | 7.3 | 0.1 | 3 | 24.3 | 1001.6 |
| 276 | 552.8 | 1100.8 |  |  | 0 | 0 | 0 | 0.569 | 282.3 | 7.3 | 0.1 | 3 | 23.8 | 997 |
| 278 | 483.7 | 963.2 |  |  | 0 | 0 | 0 | 0.569 | 282.3 | 7.3 | 0.1 | 3 | 23.5 | 1001.4 |
| 281 | 482.1 | 960 |  |  | 0 | 0 | 0 | 0.569 | 282.3 | 6.9 | 0.1 | 3 | 22.7 | 991.4 |
| 284 | 417.8 | 832 |  |  | 0 | 0 | 0 | 0.569 | 282.3 | 6.8 | 0.2 | 3 | 23.9 | 1000.3 |
| 288 | 517.4 | 1030.4 |  |  | 0 | 0 | 0 | 0.569 | 282.3 | 7.2 | 0.2 | 3 | 25.8 | 1005.3 |
| 290 | 449.9 | 1075.2 |  |  | 0 | 0 | 0 | 0.683 | 235.2 | 7.1 | 0.2 | 3 | 24.2 | 1013 |
| 292 |  |  |  |  | 0 | 0 | 0 | 0.683 | 235.2 | 7.1 | 0.1 | 3 | 23.2 | 998.8 |
| 295 |  |  |  |  | 0 | 0 | 0 | 0.683 | 235.2 | 7.1 | 0.1 | 3 | 21.5 | 1000.5 |
| 297 | 495.5 | 1184 |  |  | 0 | 0 | 0 | 0.683 | 235.2 | 7.2 | 0.1 | 3 | 21.7 | 988.8 |
| 298 | 527.6 | 1260.8 |  |  | 0 | 0 | 0 | 0.683 | 235.2 |  |  | 3 | 22.2 | 990.9 |
| 302 | 502.1 | 1200 |  |  | 0 | 0 | 0 | 0.683 | 235.2 | 6.7 | 0.2 | 3 | 22 | 990.3 |
| 306 | 447.2 | 1068.8 |  |  | 0 | 0 | 0 | 0.683 | 235.2 | 6.4 | 0.2 | 3 | 22.5 | 1003.3 |
| 309 | 520.9 | 1244.8 | 38.6 | 60.3 | 0 | 0 | 0 | 0.683 | 235.2 | 7.2 | 0.1 | 3 | 22.9 | 996 |
| 316 | 545 | 1302.4 |  |  | 0 | 0 | 0 | 0.683 | 235.2 |  |  | 3 | 21.7 | 998 |
| 323 | 480.7 | 1148.8 |  |  | 0 | 0 | 0 | 0.683 | 235.2 | 6.9 | 0.1 | 3 | 22.4 | 996.6 |
| 325 | 508.8 | 1216 |  |  | 0 | 0 | 0 | 0.683 | 235.2 | 7.1 | 0.1 | 3 | 23 | 993 |
| 330 | 531.6 | 1270.4 |  |  | 0 | 0 | 0 | 0.683 | 235.2 | 7.2 | 0.2 | 3 | 21.1 | 994 |
| 332 | 483.2 | 1347.2 |  |  | 7.4 | 0 | 0 | 0.797 | 201.6 | 7.5 | 0.2 | 3 | 21.8 | 982.3 |
| 334 | 511.9 | 1427.2 |  |  | 0 | 0 | 0 | 0.797 | 201.6 | 7.3 | 0.3 | 3 | 20.6 | 994.8 |
| 340 | 514.2 | 1433.6 |  |  | 0 | 0 | 0 | 0.797 | 201.6 | 7 | 0.2 | 3 | 20.9 | 1005 |
| 346 | 508.5 | 1417.6 | 39.5 | 59.7 | 0 | 0 | 0 | 0.797 | 201.6 | 7 | 0.1 | 3 | 22.5 | 998 |
| 348 | 523.4 | 1459.2 |  |  | 0 | 0 | 0 | 0.797 | 201.6 | 7 | 0.2 | 3 | 22.9 | 1006.8 |
| 351 | 521.1 | 1452.8 |  |  | 0 | 0 | 0 | 0.797 | 201.6 | 7.2 | 0.2 | 3 | 21 | 990.8 |
| 353 | 486.7 | 1356.8 | 38.6 | 60.3 | 0 | 0 | 0 | 0.797 | 201.6 |  |  | 3 | 23.3 | 986.3 |
| 355 | 501.6 | 1398.4 |  |  | 0 | 0 | 0 | 0.797 | 201.6 | 6.9 | 0.2 | 3 | 23.1 | 991.1 |
| 358 | 511.9 | 1427.2 |  |  |  |  |  | 0.797 | 201.6 | 7 | 0.3 | 3 | 22.4 | 1000.1 |
| 360 | 509.6 | 1420.8 |  |  | 0 | 0 | 0 | 0.797 | 201.6 | 7.2 | 0.3 | 3 | 22.2 | 990.4 |
| 362 | 507.3 | 1414.4 |  |  | 0 | 0 | 0 | 0.797 | 201.6 | 7.1 | 0.3 | 3 | 22.4 | 1011.5 |
| 365 | 524.5 | 1462.4 |  |  | 0 | 0 | 0 | 0.797 | 201.6 | 7 | 0.2 | 3 | 21.6 | 1008.8 |
| 367 | 401.7 | 1120 |  |  | 0 | 0 | 0 | 0.797 | 201.6 | 7.2 | 0.3 | 3 | 22.9 | 1008.1 |
| 372 | 484.4 | 1350.4 |  |  | 0 | 0 | 0 | 0.797 | 201.6 | 6.8 | 0.5 | 3 | 20.8 | 1004.3 |
| 374 | 516.5 | 1440 |  |  | 0 | 0 | 0 | 0.797 | 201.6 |  |  | 3 | 22.4 | 994.8 |
| 376 | 518.8 | 1446.4 |  |  | 0 | 0 | 0 | 0.797 | 201.6 | 7.2 | 0.3 | 3 | 22.2 | 999.8 |
| 379 | 529.1 | 1475.2 |  |  | 0 | 0 | 0 | 0.797 | 201.6 | 6.9 | 0.3 | 3 | 20.5 | 986.3 |
| 381 | 526.8 | 1468.8 |  |  | 0 | 0 | 0 | 0.797 | 201.6 | 7.4 | 0.3 | 3 | 22.4 | 994 |
| 383 | 506.2 | 1411.2 |  |  | 0 | 0 | 0 | 0.797 | 201.6 | 6.9 | 0.3 | 3 | 21.6 | 999.1 |
| 386 | 541.7 | 1510.4 |  |  | 0 | 0 | 0 | 0.797 | 201.6 | 6.8 | 0.4 | 3 | 22 | 993.2 |
| 388 | 503.9 | 1404.8 |  |  | 0 | 0 | 0 | 0.797 | 201.6 | 6.9 | 0.3 | 3 | 22.3 | 995 |
| 390 |  |  |  |  | 3 | 0 | 0 | 0.797 | 201.6 | 6.8 | 0.3 | 3 | 23.5 | 996.2 |
| 393 | 549.8 | 1532.8 |  |  | 0 | 0 | 0 | 0.797 | 201.6 | 7.6 | 0.3 | 3 | 21.8 | 999 |
| 395 | 538.3 | 1500.8 |  |  |  |  |  | 0.797 | 201.6 |  |  | 3 | 23.6 | 982.8 |
| 397 | 563.6 | 1571.2 |  |  | 0 | 0 | 0 | 0.797 | 201.6 | 6.7 | 0.7 | 3 | 23.3 | 993.3 |
| 400 | 519.9 | 1449.6 |  |  | 0 | 0 | 0 | 0.797 | 201.6 | 7.3 | 0.4 | 3 | 22.2 | 1001.9 |
| 404 | 524.5 | 1462.4 |  |  | 0 | 0 | 0 | 0.797 | 201.6 | 6.9 | 0.4 | 3 | 22.4 | 1009.6 |
| 407 | 519.9 | 1449.6 |  |  | 0 | 13.1 | 0 | 0.797 | 201.6 | 7.4 | 0.4 | 3 | 21 | 1010.1 |
| 409 | 505 | 1408 |  |  | 0 | 8.2 | 0 | 0.797 | 201.6 | 6.6 | 0.7 | 7 | 22.2 | 997 |
| 411 | 250.2 | 697.6 | 47.9 | 48.5 | 22.3 | 16.9 | 0 | 0.797 | 201.6 | 7.7 | 0.2 | 7 | 22.7 | 1008 |
| 414 | 284.6 | 793.6 | 60.9 | 38.2 | 23.6 | 0 | 0 | 0.797 | 201.6 | 8 | 0.1 | 7 | 21 | 997 |
| 416 | 251.4 | 700.8 |  |  | 30.9 | 0 | 0 | 0.797 | 201.6 | 8.2 | 0.2 | 7 | 21.6 | 1015.3 |
| 418 | 253.7 | 707.2 |  |  | 69.7 | 0 | 0 | 0.797 | 201.6 | 8.2 | 0.1 | 7 | 22 | 1012.7 |
| 421 | 420.1 | 1171.2 |  |  | 177.5 | 54.5 | 0 | 0.797 | 201.6 | 8.3 | 0.2 | 7 | 21.8 | 1007.2 |
| 423 | 282.4 | 787.2 |  |  | 169.1 | 38.2 | 94.4 | 0.797 | 201.6 | 8.4 | 0.1 | 7 | 23.4 | 999.3 |
| 425 | 257.1 | 716.8 |  |  | 181 | 15.9 | 85.1 | 0.797 | 201.6 | 8.4 | 0.1 | 7 | 22.6 | 989.1 |
| 428 | 213.5 | 595.2 |  |  | 60.1 | 0 | 0 | 0.797 | 201.6 | 8.6 | 0.1 | 7 | 21.5 | 985.4 |
| 431 | 486.7 | 678.4 | 69.9 | 29.2 | 32.3 | 13.5 | 0 | 0.398 | 201.6 | 8.3 | 0.1 | 3 | 22.6 | 991.1 |
| 436 | 415.5 | 579.2 |  |  | 44.2 | 0 | 0 | 0.398 | 201.6 | 8.2 | 0.1 | 3 | 22.7 | 983.3 |
| 439 | 598.3 | 834 | 48.7 | 50.3 | 52.7 | 0 | 0 | 0.398 | 201.6 | 8.1 | 0.1 | 3 | 23 | 1003 |
| 442 | 472.9 | 659.2 |  |  | 59.4 | 0 | 0 | 0.398 | 201.6 | 8.4 | 0.1 | 3 | 22.3 | 1007.7 |
| 444 | 449.9 | 627.2 |  |  | 62 | 0 | 0 | 0.398 | 201.6 | 8.4 | 0.1 | 3 | 24.1 | 1000.9 |
| 446 | 472.9 | 659.2 |  |  | 57.6 | 0 | 0 | 0.398 | 201.6 | 8.3 | 0.1 | 3 | 23.6 | 997.1 |
| 449 | 475.2 | 662.4 |  |  | 53.5 | 0 | 48.8 | 0.398 | 201.6 | 8.3 | 0.1 | 3 | 22.1 | 996.6 |
| 451 | 454.5 | 633.6 |  |  | 58.2 | 0 | 0 | 0.398 | 201.6 | 8.5 | 0.1 | 3 | 23.2 | 1000.4 |
| 456 | 463.7 | 646.4 |  |  | 47.7 | 0 | 0 | 0.398 | 201.6 | 8.2 | 0.1 | 3 | 24.9 | 1008.2 |
| 458 | 424.7 | 592 |  |  | 36.2 | 0 | 0 | 0.398 | 201.6 | 8.4 | 0.1 | 3 | 24.5 | 991.1 |
| 460 | 498.1 | 694.4 |  |  | 37.5 | 0 | 0 | 0.398 | 201.6 | 8.3 | 0.1 | 3 | 23.5 | 989 |
| 463 | 433.9 | 604.8 |  |  | 0 | 0 | 54.5 | 0.398 | 201.6 | 8.2 | 0.1 | 3 | 25.1 | 1012.6 |
| 466 | 438.4 | 611.2 |  |  | 0 | 0 | 174.2 | 0.398 | 201.6 | 8.7 | 0.2 | 3 | 26.6 | 1009.6 |
| 470 | 433.4 | 604.2 |  |  | 0 | 0 | 223 | 0.398 | 201.6 | 7.9 | 0.2 | 3 | 24.7 | 1006.7 |
| 472 | 401.7 | 560 |  |  | 0 | 0 | 0 | 0.398 | 201.6 | 8 | 0.1 | 3 | 26 | 1005.4 |
| 477 | 583.1 | 1219.2 |  |  | 0 | 0 | 231.7 | 0.597 | 201.6 | 8 | 0.1 | 3 | 23.5 | 1002.5 |
| 479 | 529.5 | 1107.2 | 39.1 | 58.2 | 0 | 0 | 0 | 0.597 | 201.6 | 8 | 0.1 | 3 | 23.6 | 1009 |
| 481 | 356.6 | 745.6 |  |  | 0 | 0 | 0 | 0.597 | 201.6 | 8.1 | 0.1 | 3 | 23.6 | 1006.9 |
| 485 | 517.3 | 1081.6 |  |  | 27.2 | 0 | 74.2 | 0.597 | 201.6 | 7.9 | 0.1 | 3 | 25.5 | 1006.9 |
| 488 |  |  |  |  | 0 | 0 | 0 | 0.597 | 201.6 | 8 | 0.1 | 3 | 25.1 | 1001.3 |
| 491 |  |  |  |  | 0 | 0 | 0 | 0.597 | 201.6 | 8 | 0.1 | 3 | 24.5 | 997 |
| 495 | 480.5 | 1004.8 |  |  | 24.8 | 0 | 70.8 | 0.597 | 201.6 | 8.1 | 0.1 | 3 | 25.4 | 984.8 |
| 502 | 498.9 | 1043.2 |  |  | 21.1 | 0 | 0 | 0.597 | 201.6 | 8.1 | 0.1 | 3 | 24.6 | 994.9 |
| 505 | 460.6 | 963.2 |  |  | 0 | 0 | 0 | 0.597 | 201.6 | 8.1 | 0.1 | 3 | 22.9 | 989 |
| 507 | 472.7 | 988.4 |  |  | 0 | 0 | 0 | 0.597 | 201.6 | 8.4 | 0.1 | 3 | 24.8 | 991.2 |
| 508 | 454.5 | 950.4 |  |  |  |  |  | 0.597 | 201.6 |  |  | 3 | 25.6 | 986.2 |
| 509 | 479 | 1001.6 |  |  | 23.5 | 0 | 0 | 0.597 | 201.6 | 8.5 | 0.1 | 3 | 24.5 | 992.5 |
| 512 | 456 | 953.6 |  |  | 6.8 | 0 | 0 | 0.597 | 201.6 | 8.4 | 0.1 | 3 | 22.8 | 1001.4 |
| 514 | 489.7 | 1024 |  |  | 4.7 | 0 | 0 | 0.597 | 201.6 | 8.4 | 0.1 | 3 | 24.8 | 1007 |
| 516 | 425.4 | 889.6 |  |  | 9.6 | 0 | 0 | 0.597 | 201.6 | 8.5 | 0.1 | 3 | 24.6 | 1009 |
| 519 | 428.5 | 896 |  |  | 6.2 | 0 | 0 | 0.597 | 201.6 | 8.2 | 0.1 | 3 | 27.1 | 997 |
| 521 | 439.2 | 918.4 |  |  | 4.2 | 0 | 0 | 0.597 | 201.6 | 8.6 | 0.1 | 3 | 25.5 | 1003 |
| 523 | 448.4 | 937.6 |  |  | 4.5 | 0 | 0 | 0.597 | 201.6 | 8.7 | 0.1 | 3 | 26.7 | 1002.8 |
| 526 | 454.5 | 950.4 |  |  | 6.1 | 0 | 0 | 0.597 | 100.8 | 8.5 | 0.1 | 3 | 27.3 | 1002.1 |

**Table S3b.** Biotic parameters. All values are given as percentages of the whole community (cells in G30, see gate template in Fig. 2d). Gates shown below summed up on average to 86.77% (± 6.11%) of all DAPI stained cells.

| Day | G1 | G2 | G3 | G4 | G5 | G6 | G8 | G9 | G10 | G15 | G16 | G17 | G20 | G21 | G22 | G25 | G26 | G27 |
| --- | --- | --- | --- | --- | --- | --- | --- | --- | --- | --- | --- | --- | --- | --- | --- | --- | --- | --- |
| 1 | 23.8 | 8.2 | 1.7 | 7.5 | 1.0 | 0.5 | 4.4 | 2.0 | 0.4 | 5.7 | 1.3 | 1.8 | 1.4 | 1.9 | 1.9 | 6.5 | 2.4 | 3.2 |
| 2 | 23.0 | 8.5 | 2.0 | 7.4 | 0.8 | 0.6 | 4.0 | 2.3 | 0.5 | 5.8 | 1.2 | 1.5 | 1.3 | 2.0 | 1.9 | 6.8 | 3.0 | 3.0 |
| 3 | 20.3 | 7.5 | 1.8 | 7.1 | 2.0 | 0.6 | 3.6 | 1.7 | 0.5 | 6.2 | 1.1 | 1.9 | 1.2 | 1.8 | 2.1 | 8.7 | 4.1 | 2.8 |
| 4 | 23.3 | 8.3 | 1.6 | 6.7 | 0.8 | 0.4 | 4.0 | 1.7 | 0.4 | 5.4 | 1.2 | 2.0 | 1.3 | 2.1 | 1.8 | 6.5 | 2.8 | 3.0 |
| 5 | 21.6 | 7.4 | 1.4 | 7.5 | 0.9 | 0.3 | 4.0 | 1.8 | 0.3 | 6.2 | 1.2 | 1.8 | 1.4 | 2.2 | 2.1 | 7.9 | 2.3 | 3.2 |
| 29 | 18.6 | 5.7 | 1.3 | 7.9 | 1.0 | 0.3 | 8.0 | 1.8 | 0.3 | 7.0 | 1.0 | 1.4 | 1.1 | 1.7 | 4.5 | 12.0 | 2.0 | 11.9 |
| 30 | 24.1 | 8.7 | 1.6 | 9.2 | 0.5 | 0.4 | 5.8 | 1.5 | 0.4 | 7.5 | 0.8 | 1.0 | 0.9 | 2.2 | 3.0 | 11.2 | 5.2 | 4.0 |
| 31 | 21.8 | 7.5 | 1.9 | 8.5 | 1.2 | 0.5 | 4.9 | 2.5 | 0.4 | 6.4 | 1.4 | 1.7 | 1.4 | 2.1 | 2.2 | 7.5 | 2.4 | 3.8 |
| 32 | 22.0 | 7.0 | 1.5 | 8.6 | 0.8 | 0.4 | 4.8 | 1.9 | 0.4 | 7.0 | 1.1 | 1.4 | 1.2 | 2.1 | 2.3 | 8.9 | 2.4 | 4.3 |
| 33 | 21.6 | 7.8 | 1.9 | 9.1 | 1.2 | 0.5 | 4.8 | 2.1 | 0.4 | 6.3 | 1.2 | 1.7 | 1.3 | 2.1 | 2.0 | 6.6 | 2.4 | 3.5 |
| 38 | 19.0 | 8.7 | 2.1 | 9.5 | 1.1 | 0.5 | 3.5 | 2.9 | 0.5 | 7.6 | 1.7 | 1.7 | 1.8 | 2.2 | 2.1 | 7.7 | 3.3 | 2.4 |
| 45 | 20.8 | 7.4 | 1.6 | 8.1 | 1.4 | 0.4 | 5.2 | 2.0 | 0.3 | 6.6 | 1.3 | 1.7 | 1.4 | 1.7 | 2.4 | 8.0 | 2.6 | 3.4 |
| 57 | 19.7 | 8.6 | 2.3 | 7.9 | 1.8 | 0.6 | 5.0 | 3.1 | 0.5 | 6.4 | 1.7 | 2.1 | 1.7 | 1.5 | 2.8 | 7.0 | 2.9 | 3.0 |
| 59 | 17.7 | 7.4 | 1.6 | 12.6 | 1.1 | 0.4 | 4.4 | 1.5 | 0.4 | 11.5 | 1.2 | 1.5 | 1.2 | 2.0 | 5.7 | 11.7 | 3.3 | 4.3 |
| 61 | 17.5 | 6.9 | 1.4 | 12.1 | 1.6 | 0.3 | 6.0 | 1.6 | 0.3 | 10.9 | 1.2 | 1.6 | 1.3 | 1.9 | 5.5 | 11.3 | 3.3 | 4.3 |
| 64 | 17.0 | 6.9 | 1.6 | 13.2 | 1.0 | 0.4 | 4.6 | 1.6 | 0.4 | 13.6 | 1.0 | 1.3 | 1.1 | 2.0 | 7.5 | 14.1 | 3.6 | 5.2 |
| 66 | 21.3 | 8.1 | 1.6 | 10.5 | 1.3 | 0.4 | 4.6 | 1.5 | 0.3 | 7.9 | 1.1 | 1.6 | 1.1 | 1.6 | 2.5 | 7.7 | 2.6 | 3.2 |
| 68 | 22.2 | 9.2 | 1.6 | 11.2 | 1.4 | 0.4 | 5.3 | 1.3 | 0.3 | 10.3 | 1.1 | 1.5 | 1.1 | 2.1 | 3.0 | 7.6 | 3.1 | 3.0 |
| 71 | 21.6 | 8.6 | 1.4 | 12.6 | 0.9 | 0.3 | 5.6 | 1.2 | 0.3 | 11.9 | 0.9 | 1.1 | 0.9 | 2.1 | 3.6 | 9.0 | 2.9 | 3.5 |
| 74 | 19.7 | 6.5 | 1.3 | 7.3 | 1.6 | 0.3 | 17.2 | 1.2 | 0.2 | 4.8 | 1.0 | 1.3 | 1.1 | 2.0 | 1.9 | 5.0 | 4.3 | 3.8 |
| 79 | 28.0 | 6.6 | 1.0 | 5.5 | 0.4 | 0.3 | 22.6 | 0.9 | 0.3 | 4.5 | 0.4 | 0.6 | 0.5 | 2.0 | 2.0 | 7.1 | 3.8 | 5.4 |
| 80 | 26.1 | 6.8 | 1.2 | 6.7 | 0.6 | 0.3 | 18.7 | 1.0 | 0.3 | 5.2 | 0.6 | 0.8 | 0.6 | 2.0 | 2.1 | 7.2 | 3.3 | 5.0 |
| 81 | 22.9 | 6.8 | 1.2 | 7.2 | 1.2 | 0.3 | 16.3 | 1.5 | 0.3 | 5.2 | 1.0 | 1.4 | 1.2 | 1.9 | 2.1 | 6.4 | 3.2 | 4.4 |
| 82 | 24.7 | 5.9 | 1.0 | 6.2 | 0.7 | 0.2 | 21.6 | 1.1 | 0.3 | 4.7 | 0.7 | 0.9 | 0.7 | 1.9 | 2.0 | 7.0 | 3.5 | 5.4 |
| 87 | 21.9 | 8.9 | 1.7 | 11.3 | 0.9 | 0.4 | 7.0 | 1.7 | 0.4 | 9.4 | 1.1 | 1.3 | 1.2 | 1.4 | 3.9 | 12.2 | 3.3 | 4.7 |
| 89 | 22.7 | 9.4 | 2.1 | 7.3 | 1.3 | 0.5 | 7.6 | 2.3 | 0.5 | 8.5 | 1.4 | 1.3 | 1.5 | 1.7 | 3.7 | 11.4 | 2.6 | 4.7 |
| 92 | 19.7 | 9.1 | 2.0 | 11.0 | 1.1 | 0.4 | 6.2 | 2.7 | 0.5 | 9.3 | 1.6 | 1.7 | 1.6 | 1.3 | 3.6 | 10.9 | 3.2 | 4.1 |
| 93 | 16.0 | 8.5 | 2.6 | 8.1 | 2.5 | 0.6 | 3.3 | 4.8 | 0.7 | 7.2 | 3.1 | 3.3 | 3.0 | 1.0 | 2.3 | 5.2 | 2.3 | 2.0 |
| 94 | 16.6 | 9.6 | 2.7 | 8.3 | 1.2 | 0.7 | 3.5 | 4.2 | 0.9 | 7.1 | 2.5 | 2.4 | 2.5 | 1.5 | 2.2 | 6.5 | 3.6 | 2.2 |
| 97 | 25.9 | 16.8 | 6.3 | 4.6 | 1.1 | 2.4 | 2.9 | 3.9 | 2.6 | 4.4 | 0.9 | 0.8 | 1.0 | 1.8 | 2.0 | 5.4 | 5.9 | 2.3 |
| 113 | 19.2 | 8.1 | 2.0 | 9.3 | 2.2 | 0.4 | 5.5 | 3.1 | 0.5 | 7.3 | 2.1 | 2.4 | 2.2 | 0.9 | 2.8 | 6.7 | 2.4 | 3.1 |
| 115 | 20.3 | 8.5 | 2.1 | 8.4 | 1.8 | 0.5 | 4.8 | 3.5 | 0.5 | 7.0 | 2.2 | 2.5 | 2.3 | 0.7 | 2.9 | 7.0 | 2.1 | 3.1 |
| 120 | 18.1 | 8.9 | 2.2 | 10.4 | 1.2 | 0.5 | 4.3 | 3.3 | 0.6 | 8.9 | 2.2 | 2.1 | 2.2 | 1.2 | 2.8 | 6.8 | 3.3 | 2.7 |
| 122 | 19.1 | 8.1 | 2.2 | 8.6 | 2.2 | 0.5 | 4.4 | 3.9 | 0.6 | 6.7 | 2.5 | 2.8 | 2.6 | 0.8 | 2.6 | 6.0 | 2.2 | 2.6 |
| 129 | 19.5 | 8.2 | 2.1 | 8.1 | 2.1 | 0.5 | 5.1 | 3.5 | 0.5 | 6.8 | 2.2 | 2.5 | 2.3 | 1.0 | 2.8 | 7.1 | 2.4 | 3.1 |
| 131 | 27.0 | 9.2 | 1.7 | 7.1 | 0.4 | 0.4 | 6.1 | 1.6 | 0.4 | 8.2 | 0.7 | 0.6 | 0.7 | 1.4 | 3.9 | 14.4 | 3.1 | 5.2 |
| 134 | 26.0 | 10.4 | 2.1 | 10.5 | 0.6 | 0.6 | 6.3 | 2.2 | 0.6 | 8.5 | 0.9 | 0.9 | 0.8 | 1.6 | 3.6 | 11.8 | 3.2 | 4.5 |
| 136 | 18.8 | 9.2 | 2.7 | 9.7 | 1.4 | 0.6 | 4.6 | 4.3 | 0.9 | 8.1 | 2.1 | 2.0 | 2.0 | 1.3 | 2.8 | 7.5 | 2.7 | 2.9 |
| 138 | 18.8 | 9.2 | 2.7 | 9.7 | 1.4 | 0.6 | 4.6 | 4.3 | 0.9 | 8.1 | 2.1 | 2.0 | 2.0 | 1.3 | 2.8 | 7.5 | 2.7 | 2.9 |
| 143 | 16.5 | 8.9 | 2.4 | 11.2 | 1.1 | 0.6 | 3.6 | 3.2 | 0.7 | 9.3 | 2.1 | 2.3 | 2.3 | 1.5 | 2.4 | 6.5 | 3.7 | 2.2 |
| 145 | 17.2 | 8.8 | 2.7 | 6.6 | 2.4 | 0.7 | 4.6 | 4.7 | 0.9 | 5.4 | 2.6 | 3.1 | 2.8 | 0.8 | 2.7 | 4.9 | 2.5 | 2.3 |
| 152 | 25.8 | 7.7 | 2.1 | 7.0 | 1.4 | 0.5 | 4.7 | 2.7 | 0.6 | 5.8 | 1.6 | 1.7 | 1.5 | 2.8 | 1.9 | 4.4 | 6.7 | 2.2 |
| 155 | 30.0 | 8.2 | 2.1 | 6.5 | 0.9 | 0.5 | 5.2 | 2.2 | 0.6 | 5.3 | 1.0 | 1.1 | 0.9 | 3.2 | 2.1 | 5.5 | 7.6 | 2.7 |
| 157 | 24.5 | 8.0 | 2.1 | 7.0 | 1.7 | 0.5 | 4.8 | 2.8 | 0.7 | 5.8 | 1.6 | 1.8 | 1.6 | 2.6 | 2.0 | 4.6 | 6.0 | 2.3 |
| 159 | 33.5 | 7.8 | 1.6 | 6.5 | 0.8 | 0.4 | 6.2 | 1.2 | 0.4 | 6.0 | 0.7 | 0.9 | 0.7 | 3.2 | 2.7 | 7.8 | 6.7 | 3.5 |
| 162 | 33.6 | 8.2 | 1.6 | 5.8 | 0.6 | 0.5 | 5.5 | 0.8 | 0.5 | 5.4 | 0.5 | 0.7 | 0.5 | 3.8 | 2.4 | 7.0 | 7.8 | 3.1 |
| 164 | 13.7 | 10.4 | 2.3 | 7.0 | 1.5 | 0.8 | 12.6 | 2.6 | 0.6 | 5.1 | 1.7 | 1.8 | 1.9 | 1.2 | 2.7 | 5.8 | 3.4 | 3.8 |
| 166 | 24.3 | 11.9 | 2.6 | 6.2 | 0.7 | 0.8 | 3.7 | 2.2 | 0.9 | 4.6 | 0.9 | 1.0 | 0.9 | 2.1 | 1.8 | 5.9 | 3.3 | 2.2 |
| 170 | 21.4 | 11.8 | 3.4 | 6.8 | 1.6 | 1.2 | 4.7 | 4.0 | 1.3 | 5.3 | 1.7 | 2.1 | 1.8 | 1.7 | 2.1 | 5.1 | 3.6 | 2.5 |
| 173 | 23.1 | 11.6 | 2.9 | 6.7 | 0.5 | 0.9 | 3.8 | 2.6 | 1.2 | 5.1 | 0.9 | 0.9 | 0.8 | 1.3 | 2.3 | 7.1 | 3.3 | 2.7 |
| 176 | 22.4 | 12.0 | 3.1 | 6.8 | 0.7 | 1.0 | 3.6 | 3.6 | 1.4 | 5.4 | 1.1 | 1.1 | 1.0 | 1.2 | 2.4 | 6.2 | 3.9 | 2.5 |
| 178 | 11.1 | 12.6 | 2.9 | 4.7 | 2.0 | 0.8 | 6.2 | 4.2 | 0.7 | 5.2 | 2.9 | 2.2 | 3.4 | 0.6 | 3.1 | 6.0 | 2.6 | 3.5 |
| 180 | 22.5 | 12.5 | 3.5 | 5.0 | 1.3 | 1.2 | 5.6 | 4.1 | 1.5 | 4.2 | 1.6 | 2.0 | 1.8 | 1.8 | 1.7 | 4.7 | 4.5 | 2.4 |
| 183 | 26.5 | 14.9 | 3.6 | 5.4 | 0.3 | 1.2 | 3.7 | 2.5 | 1.6 | 4.1 | 0.5 | 0.5 | 0.4 | 1.8 | 2.1 | 7.1 | 3.5 | 2.7 |
| 187 | 25.3 | 13.7 | 3.4 | 5.3 | 0.3 | 1.1 | 4.5 | 3.2 | 1.6 | 4.2 | 0.6 | 0.8 | 0.6 | 1.7 | 2.2 | 6.7 | 3.7 | 2.4 |
| 190 | 27.7 | 16.3 | 4.2 | 4.4 | 0.3 | 1.5 | 3.0 | 3.6 | 2.1 | 3.9 | 0.6 | 0.8 | 0.6 | 2.0 | 2.2 | 6.2 | 3.6 | 2.3 |
| 192 | 32.0 | 17.5 | 3.7 | 4.9 | 0.2 | 1.4 | 4.6 | 1.6 | 1.5 | 4.9 | 0.2 | 0.7 | 0.2 | 2.2 | 3.3 | 10.5 | 4.3 | 3.7 |
| 194 | 24.2 | 16.2 | 4.8 | 3.6 | 0.5 | 1.8 | 2.7 | 4.1 | 2.5 | 3.1 | 0.8 | 1.0 | 0.7 | 2.3 | 1.6 | 4.8 | 5.9 | 1.9 |
| 199 | 23.7 | 15.9 | 4.8 | 3.7 | 0.9 | 1.6 | 4.2 | 4.7 | 2.1 | 4.5 | 1.5 | 0.9 | 1.6 | 1.1 | 2.1 | 4.9 | 3.3 | 2.2 |
| 201 | 26.6 | 19.2 | 5.4 | 4.1 | 0.6 | 2.0 | 3.4 | 3.5 | 2.2 | 5.1 | 0.9 | 0.6 | 0.9 | 1.7 | 2.1 | 5.7 | 3.7 | 2.1 |
| 204 | 27.9 | 21.4 | 5.6 | 3.6 | 0.3 | 2.1 | 4.8 | 2.0 | 2.3 | 5.2 | 0.4 | 0.2 | 0.4 | 2.8 | 2.1 | 6.7 | 4.2 | 2.4 |
| 206 | 24.2 | 18.4 | 5.9 | 2.9 | 0.7 | 2.1 | 4.7 | 3.9 | 2.6 | 3.9 | 0.9 | 0.6 | 1.0 | 2.5 | 1.7 | 4.9 | 4.5 | 2.0 |
| 208 | 27.0 | 21.4 | 5.3 | 3.5 | 0.2 | 2.0 | 3.9 | 2.4 | 2.3 | 3.5 | 0.3 | 0.8 | 0.3 | 2.8 | 2.3 | 6.7 | 6.0 | 2.4 |
| 211 | 25.0 | 19.9 | 5.4 | 3.8 | 0.4 | 2.1 | 3.1 | 2.6 | 2.4 | 3.8 | 0.6 | 0.9 | 0.5 | 2.6 | 2.3 | 6.6 | 6.2 | 2.2 |
| 212 | 26.2 | 20.4 | 5.3 | 4.2 | 0.3 | 2.0 | 3.7 | 2.7 | 2.4 | 3.9 | 0.5 | 0.9 | 0.4 | 2.3 | 2.1 | 6.4 | 5.4 | 2.3 |
| 213 | 26.2 | 20.4 | 5.3 | 4.2 | 0.3 | 2.0 | 3.7 | 2.7 | 2.4 | 3.9 | 0.5 | 0.9 | 0.4 | 2.3 | 2.1 | 6.4 | 5.4 | 2.3 |
| 215 | 15.8 | 15.8 | 5.7 | 3.1 | 0.4 | 2.4 | 2.5 | 4.7 | 3.4 | 2.7 | 0.9 | 1.0 | 0.8 | 2.7 | 1.4 | 3.9 | 7.5 | 1.7 |
| 218 | 21.5 | 18.3 | 5.9 | 2.0 | 0.5 | 2.5 | 3.6 | 4.8 | 3.2 | 2.3 | 0.8 | 0.6 | 0.6 | 1.8 | 1.6 | 4.4 | 5.1 | 2.1 |
| 220 | 17.8 | 14.7 | 4.5 | 3.8 | 1.1 | 1.6 | 4.6 | 4.7 | 1.9 | 2.6 | 1.5 | 1.4 | 1.4 | 1.7 | 1.6 | 4.1 | 4.2 | 2.2 |
| 222 | 16.1 | 12.1 | 3.9 | 3.9 | 1.1 | 1.3 | 10.0 | 4.4 | 1.9 | 3.4 | 1.3 | 1.1 | 1.2 | 2.0 | 2.5 | 5.7 | 6.7 | 3.3 |
| 225 | 17.7 | 13.7 | 4.0 | 3.6 | 0.8 | 1.4 | 9.4 | 4.2 | 1.9 | 3.2 | 1.1 | 1.1 | 1.0 | 2.0 | 2.4 | 5.6 | 6.6 | 2.9 |
| 229 | 19.1 | 14.6 | 3.6 | 4.2 | 0.9 | 1.2 | 8.6 | 2.6 | 1.2 | 3.7 | 1.0 | 1.4 | 0.9 | 2.1 | 3.0 | 7.0 | 4.9 | 3.6 |
| 232 | 17.3 | 12.7 | 3.4 | 4.3 | 0.7 | 1.2 | 9.5 | 2.9 | 1.4 | 3.8 | 0.9 | 0.8 | 0.9 | 2.9 | 3.4 | 8.7 | 5.3 | 5.3 |
| 234 | 24.2 | 14.5 | 3.0 | 4.4 | 0.4 | 1.1 | 4.3 | 2.4 | 1.1 | 6.4 | 0.9 | 0.9 | 1.0 | 4.5 | 2.9 | 8.9 | 4.4 | 3.2 |
| 236 | 24.0 | 14.7 | 3.1 | 5.3 | 0.5 | 1.1 | 3.9 | 2.6 | 1.0 | 6.4 | 0.9 | 1.0 | 1.0 | 4.0 | 2.6 | 7.6 | 4.5 | 2.9 |
| 239 | 25.7 | 15.8 | 3.3 | 4.8 | 1.4 | 1.2 | 3.9 | 2.5 | 1.1 | 5.9 | 0.8 | 0.8 | 0.9 | 3.7 | 2.5 | 6.9 | 5.6 | 2.7 |
| 241 | 22.6 | 13.4 | 2.8 | 4.4 | 2.0 | 0.9 | 3.9 | 3.4 | 0.9 | 4.9 | 1.5 | 1.6 | 1.7 | 2.8 | 2.0 | 5.7 | 3.9 | 2.6 |
| 243 | 26.9 | 16.7 | 3.5 | 4.4 | 1.3 | 1.3 | 4.1 | 2.4 | 1.1 | 6.0 | 0.8 | 0.8 | 0.9 | 3.6 | 2.6 | 7.4 | 5.3 | 2.8 |
| 246 | 28.0 | 17.0 | 3.3 | 4.0 | 1.1 | 1.4 | 4.5 | 2.2 | 1.2 | 5.9 | 0.7 | 0.7 | 0.8 | 4.8 | 2.7 | 8.1 | 6.7 | 3.1 |
| 248 | 14.1 | 19.6 | 5.6 | 5.3 | 1.4 | 1.9 | 4.7 | 4.3 | 1.7 | 4.4 | 1.0 | 1.1 | 1.2 | 1.1 | 3.2 | 6.3 | 3.6 | 2.9 |
| 250 | 27.4 | 17.3 | 3.4 | 4.1 | 1.0 | 1.4 | 4.5 | 2.0 | 1.2 | 6.2 | 0.7 | 0.7 | 0.8 | 4.8 | 2.7 | 8.8 | 6.7 | 3.1 |
| 253 | 26.4 | 16.0 | 3.0 | 4.7 | 1.1 | 1.2 | 4.7 | 2.1 | 1.0 | 6.5 | 0.7 | 0.8 | 0.8 | 4.4 | 2.8 | 8.7 | 6.1 | 3.3 |
| 256 | 23.9 | 15.0 | 2.8 | 4.8 | 1.2 | 1.1 | 4.6 | 2.0 | 1.0 | 6.4 | 0.8 | 1.0 | 1.0 | 4.1 | 2.7 | 8.9 | 5.8 | 3.2 |
| 260 | 25.6 | 16.1 | 3.3 | 4.2 | 0.9 | 1.3 | 4.5 | 2.2 | 1.4 | 6.2 | 0.6 | 0.7 | 0.7 | 4.7 | 2.7 | 8.7 | 6.5 | 3.4 |
| 262 | 21.0 | 14.3 | 4.1 | 6.9 | 1.8 | 1.3 | 4.0 | 3.8 | 1.3 | 6.0 | 1.1 | 1.7 | 1.6 | 1.1 | 3.0 | 6.0 | 2.6 | 2.4 |
| 264 | 17.8 | 11.9 | 3.5 | 7.1 | 2.6 | 1.0 | 4.2 | 4.0 | 0.9 | 6.4 | 1.6 | 2.4 | 2.1 | 0.8 | 3.3 | 6.4 | 2.2 | 2.6 |
| 267 | 19.6 | 14.4 | 3.8 | 6.9 | 1.6 | 1.2 | 4.4 | 3.2 | 1.0 | 6.3 | 1.1 | 1.5 | 1.4 | 0.8 | 3.2 | 7.0 | 2.9 | 2.7 |
| 269 | 22.6 | 18.0 | 3.7 | 4.3 | 0.5 | 1.4 | 4.0 | 2.5 | 1.3 | 6.3 | 0.8 | 0.8 | 0.8 | 4.7 | 2.9 | 8.6 | 5.2 | 3.7 |
| 271 | 21.8 | 16.3 | 3.4 | 3.7 | 1.1 | 1.4 | 3.8 | 2.7 | 1.3 | 5.3 | 0.9 | 0.8 | 1.1 | 5.0 | 2.6 | 7.5 | 8.2 | 3.2 |
| 274 | 22.0 | 16.1 | 3.7 | 3.4 | 1.0 | 1.6 | 3.6 | 3.0 | 1.4 | 4.2 | 1.0 | 0.7 | 1.1 | 7.2 | 2.1 | 6.1 | 9.9 | 3.2 |
| 276 | 24.1 | 18.5 | 4.1 | 3.4 | 0.6 | 1.8 | 3.7 | 2.3 | 1.8 | 6.4 | 0.5 | 0.5 | 0.6 | 4.8 | 3.2 | 9.7 | 9.4 | 3.6 |
| 278 | 25.9 | 20.0 | 4.0 | 3.7 | 0.8 | 1.6 | 3.3 | 2.2 | 1.6 | 5.7 | 0.6 | 0.6 | 0.7 | 4.2 | 2.8 | 7.8 | 7.4 | 3.1 |
| 281 | 28.6 | 25.2 | 4.9 | 2.4 | 0.3 | 2.5 | 2.6 | 1.3 | 2.6 | 4.9 | 0.2 | 0.1 | 0.3 | 3.8 | 2.4 | 7.5 | 9.3 | 2.7 |
| 284 | 30.7 | 27.2 | 4.4 | 2.2 | 0.1 | 1.7 | 2.1 | 1.2 | 1.7 | 3.9 | 0.2 | 0.3 | 0.3 | 3.9 | 1.9 | 5.5 | 6.2 | 2.1 |
| 288 | 23.0 | 23.9 | 4.3 | 2.3 | 0.3 | 2.0 | 1.9 | 1.8 | 2.2 | 3.2 | 0.5 | 0.5 | 0.6 | 6.0 | 1.6 | 4.3 | 8.2 | 1.9 |
| 290 | 26.0 | 16.4 | 3.4 | 3.5 | 1.3 | 1.2 | 3.2 | 3.0 | 1.3 | 3.7 | 0.9 | 0.8 | 1.0 | 4.1 | 1.5 | 4.8 | 4.2 | 2.5 |
| 292 | 29.4 | 18.0 | 3.9 | 2.4 | 0.9 | 1.6 | 2.9 | 2.8 | 1.6 | 2.9 | 0.7 | 0.5 | 0.7 | 7.6 | 1.3 | 4.1 | 7.0 | 2.5 |
| 295 | 26.9 | 20.9 | 3.9 | 3.1 | 1.0 | 1.6 | 2.5 | 2.8 | 1.6 | 3.2 | 0.9 | 0.6 | 0.9 | 5.4 | 1.3 | 3.9 | 6.7 | 1.6 |
| 297 | 32.1 | 27.7 | 4.5 | 1.4 | 0.5 | 2.2 | 1.6 | 1.3 | 2.2 | 2.0 | 0.3 | 0.3 | 0.4 | 4.2 | 1.1 | 2.8 | 6.1 | 1.1 |
| 298 | 33.5 | 27.4 | 4.4 | 1.5 | 0.6 | 2.1 | 1.5 | 1.7 | 2.0 | 1.8 | 0.5 | 0.4 | 0.6 | 3.8 | 0.8 | 2.2 | 5.2 | 1.1 |
| 302 | 39.1 | 31.7 | 4.5 | 0.7 | 0.3 | 2.1 | 1.2 | 0.8 | 2.1 | 1.2 | 0.2 | 0.1 | 0.2 | 3.5 | 0.6 | 1.6 | 5.1 | 0.8 |
| 306 | 37.8 | 29.2 | 4.6 | 0.9 | 0.3 | 2.4 | 1.4 | 0.8 | 2.2 | 1.5 | 0.2 | 0.1 | 0.2 | 5.7 | 0.8 | 2.1 | 6.2 | 0.8 |
| 309 | 26.2 | 21.7 | 3.5 | 2.6 | 0.3 | 1.2 | 2.1 | 2.0 | 1.2 | 2.7 | 0.7 | 0.6 | 0.8 | 5.1 | 1.2 | 3.5 | 5.8 | 1.8 |
| 316 | 29.2 | 23.6 | 5.5 | 1.4 | 0.7 | 2.5 | 1.7 | 3.2 | 3.1 | 1.9 | 0.8 | 0.9 | 1.1 | 2.9 | 1.0 | 2.4 | 4.3 | 1.1 |
| 323 | 19.0 | 17.2 | 5.5 | 2.6 | 0.4 | 1.9 | 1.8 | 6.0 | 3.1 | 2.7 | 1.7 | 1.5 | 1.8 | 2.5 | 1.2 | 3.0 | 4.4 | 1.4 |
| 325 | 26.6 | 29.5 | 6.7 | 1.0 | 0.1 | 2.5 | 1.4 | 2.2 | 3.1 | 1.6 | 0.5 | 0.4 | 0.5 | 2.9 | 0.8 | 2.1 | 5.0 | 0.9 |
| 330 | 25.6 | 30.6 | 8.0 | 0.7 | 0.1 | 3.0 | 1.2 | 1.6 | 3.6 | 1.4 | 0.2 | 0.2 | 0.3 | 1.9 | 0.7 | 2.1 | 5.8 | 0.9 |
| 332 | 25.5 | 31.1 | 6.7 | 1.8 | 0.1 | 2.0 | 2.0 | 1.6 | 1.9 | 2.6 | 0.5 | 0.4 | 0.6 | 2.2 | 1.2 | 3.5 | 5.6 | 1.3 |
| 334 | 24.2 | 26.9 | 7.3 | 2.1 | 0.3 | 3.0 | 2.0 | 1.4 | 2.7 | 3.0 | 0.4 | 0.3 | 0.5 | 5.6 | 1.4 | 4.5 | 6.2 | 1.6 |
| 340 | 23.6 | 25.0 | 7.3 | 1.8 | 0.8 | 2.4 | 2.2 | 2.4 | 2.2 | 2.4 | 1.1 | 0.8 | 1.4 | 5.6 | 1.2 | 3.4 | 5.9 | 1.6 |
| 346 | 23.9 | 29.3 | 8.2 | 1.3 | 0.4 | 3.3 | 1.6 | 1.9 | 3.2 | 2.0 | 0.6 | 0.5 | 0.8 | 4.6 | 1.1 | 2.7 | 5.7 | 1.2 |
| 348 | 26.1 | 33.2 | 7.8 | 0.7 | 0.2 | 3.4 | 1.5 | 0.8 | 3.0 | 1.6 | 0.3 | 0.2 | 0.4 | 5.3 | 0.9 | 2.5 | 5.7 | 1.1 |
| 351 | 21.1 | 21.9 | 6.2 | 2.1 | 0.6 | 2.8 | 2.0 | 3.2 | 3.0 | 2.8 | 0.9 | 0.7 | 1.3 | 6.2 | 1.4 | 3.9 | 6.8 | 1.6 |
| 353 | 25.6 | 25.9 | 5.9 | 1.6 | 0.4 | 2.5 | 2.1 | 2.0 | 2.5 | 3.0 | 0.6 | 0.5 | 0.8 | 5.6 | 1.7 | 4.6 | 5.8 | 1.9 |
| 355 | 27.4 | 31.9 | 6.9 | 0.8 | 0.2 | 3.1 | 1.4 | 0.7 | 3.0 | 1.6 | 0.2 | 0.2 | 0.3 | 3.6 | 0.9 | 2.8 | 6.3 | 1.3 |
| 358 | 26.4 | 22.5 | 4.5 | 2.8 | 1.1 | 1.6 | 1.8 | 3.8 | 1.8 | 2.3 | 1.9 | 2.4 | 2.1 | 5.4 | 0.9 | 2.6 | 4.0 | 1.0 |
| 360 | 27.0 | 21.8 | 4.2 | 2.6 | 1.0 | 1.3 | 2.4 | 3.3 | 1.3 | 2.3 | 1.7 | 2.2 | 1.8 | 4.6 | 1.6 | 4.2 | 3.6 | 1.8 |
| 362 | 28.7 | 24.8 | 6.2 | 1.5 | 0.5 | 2.1 | 1.7 | 2.5 | 2.6 | 1.9 | 0.9 | 0.9 | 1.3 | 3.1 | 0.8 | 2.7 | 7.2 | 0.9 |
| 365 | 29.0 | 25.7 | 5.9 | 1.3 | 0.5 | 2.0 | 1.6 | 2.2 | 2.3 | 1.7 | 0.9 | 0.9 | 1.4 | 2.7 | 0.7 | 2.4 | 7.4 | 0.8 |
| 367 | 26.9 | 27.2 | 6.2 | 1.1 | 0.2 | 2.2 | 1.2 | 1.3 | 2.6 | 1.5 | 0.4 | 0.3 | 0.8 | 3.2 | 0.6 | 2.2 | 8.7 | 0.8 |
| 372 | 26.2 | 30.3 | 7.1 | 0.9 | 0.1 | 2.6 | 1.5 | 0.8 | 3.2 | 1.5 | 0.2 | 0.3 | 0.4 | 3.1 | 0.7 | 2.2 | 4.8 | 1.0 |
| 374 | 26.9 | 21.4 | 4.0 | 2.8 | 0.7 | 1.2 | 3.5 | 3.7 | 1.4 | 2.7 | 1.3 | 1.9 | 1.5 | 2.1 | 1.2 | 2.8 | 4.4 | 1.4 |
| 376 | 24.0 | 33.4 | 8.4 | 1.2 | 0.2 | 2.6 | 1.7 | 1.1 | 3.1 | 1.7 | 0.3 | 0.2 | 0.5 | 2.3 | 0.9 | 2.1 | 5.2 | 1.1 |
| 379 | 20.3 | 23.8 | 6.2 | 2.5 | 0.3 | 1.7 | 2.6 | 4.0 | 2.1 | 2.9 | 1.1 | 1.0 | 1.3 | 1.8 | 1.3 | 3.6 | 6.9 | 1.5 |
| 381 | 25.3 | 26.5 | 5.7 | 2.5 | 0.7 | 1.7 | 2.8 | 3.0 | 1.9 | 2.8 | 1.1 | 1.1 | 1.6 | 1.9 | 1.3 | 2.9 | 5.6 | 1.6 |
| 383 | 23.5 | 27.7 | 6.4 | 1.6 | 0.1 | 2.4 | 1.5 | 2.0 | 2.4 | 1.9 | 0.8 | 0.8 | 1.0 | 3.4 | 1.0 | 2.9 | 5.2 | 1.3 |
| 386 | 23.0 | 26.1 | 8.8 | 2.2 | 0.4 | 2.3 | 2.8 | 2.5 | 2.7 | 2.5 | 0.5 | 0.3 | 0.7 | 1.7 | 1.2 | 2.5 | 9.4 | 1.3 |
| 388 | 22.9 | 31.6 | 8.9 | 1.6 | 0.2 | 2.7 | 2.1 | 1.4 | 3.3 | 1.9 | 0.2 | 0.1 | 0.3 | 2.2 | 1.0 | 1.9 | 7.2 | 1.1 |
| 390 | 21.8 | 31.7 | 6.3 | 1.9 | 0.1 | 2.1 | 2.6 | 2.3 | 2.5 | 2.1 | 0.4 | 0.3 | 0.5 | 2.4 | 1.2 | 2.0 | 6.8 | 1.1 |
| 393 | 24.3 | 16.7 | 4.9 | 2.0 | 1.0 | 1.5 | 4.0 | 4.0 | 1.9 | 2.9 | 1.2 | 0.6 | 1.5 | 1.0 | 1.6 | 4.2 | 7.2 | 2.3 |
| 395 | 19.9 | 28.1 | 10.5 | 2.3 | 0.2 | 2.5 | 3.5 | 1.4 | 3.0 | 2.8 | 0.3 | 0.2 | 0.4 | 2.3 | 1.5 | 2.9 | 7.9 | 1.8 |
| 397 | 25.7 | 22.6 | 6.8 | 2.0 | 0.9 | 1.9 | 4.6 | 2.9 | 2.3 | 2.5 | 0.6 | 0.5 | 0.9 | 1.0 | 1.7 | 2.7 | 7.6 | 2.2 |
| 400 | 25.0 | 20.6 | 5.1 | 2.0 | 1.1 | 1.5 | 5.4 | 3.3 | 1.7 | 2.8 | 1.0 | 0.7 | 1.5 | 1.0 | 1.9 | 3.3 | 5.8 | 2.9 |
| 404 | 26.1 | 23.7 | 5.4 | 1.7 | 0.7 | 1.9 | 4.6 | 2.6 | 2.2 | 2.6 | 0.7 | 0.4 | 1.1 | 0.9 | 1.8 | 2.9 | 7.0 | 2.4 |
| 407 | 23.0 | 31.7 | 7.2 | 1.4 | 0.1 | 2.9 | 2.5 | 2.2 | 3.1 | 2.0 | 0.5 | 0.3 | 0.6 | 2.0 | 1.3 | 2.2 | 6.2 | 1.3 |
| 409 | 19.3 | 20.1 | 5.4 | 2.1 | 0.3 | 1.8 | 4.7 | 3.9 | 2.0 | 3.5 | 1.2 | 0.9 | 1.6 | 1.5 | 2.4 | 4.2 | 6.9 | 2.6 |
| 411 | 23.9 | 20.0 | 6.6 | 2.0 | 0.9 | 2.0 | 4.5 | 3.4 | 2.3 | 3.2 | 0.9 | 0.5 | 1.2 | 1.0 | 2.1 | 3.9 | 6.5 | 2.8 |
| 414 | 28.0 | 24.9 | 7.2 | 1.8 | 0.8 | 2.0 | 2.4 | 2.4 | 2.4 | 3.0 | 0.6 | 0.3 | 0.7 | 1.3 | 2.0 | 3.7 | 4.9 | 2.0 |
| 416 | 27.4 | 22.6 | 6.7 | 1.6 | 0.8 | 1.9 | 1.7 | 3.4 | 2.6 | 2.5 | 0.9 | 0.5 | 1.2 | 1.0 | 1.5 | 3.1 | 6.5 | 1.3 |
| 418 | 25.1 | 26.2 | 6.4 | 1.4 | 3.3 | 2.0 | 1.5 | 4.0 | 2.6 | 1.9 | 2.1 | 0.8 | 1.7 | 1.5 | 1.0 | 2.0 | 5.3 | 0.8 |
| 421 | 16.6 | 8.0 | 1.5 | 12.3 | 5.4 | 0.4 | 5.1 | 3.9 | 0.4 | 10.7 | 3.6 | 2.1 | 3.4 | 0.9 | 2.5 | 6.1 | 2.7 | 2.5 |
| 423 | 21.6 | 9.0 | 2.1 | 10.9 | 6.2 | 0.5 | 3.4 | 7.1 | 0.7 | 10.8 | 3.3 | 1.7 | 2.7 | 0.7 | 2.5 | 7.6 | 3.9 | 2.0 |
| 425 | 16.1 | 8.5 | 1.8 | 11.3 | 4.1 | 0.4 | 2.8 | 5.5 | 0.5 | 11.5 | 3.5 | 2.5 | 3.7 | 0.9 | 2.8 | 9.3 | 3.1 | 1.7 |
| 428 | 17.4 | 9.6 | 2.3 | 9.7 | 5.1 | 0.6 | 2.1 | 6.5 | 0.8 | 9.1 | 3.7 | 2.4 | 3.5 | 1.3 | 2.0 | 6.3 | 3.8 | 1.3 |
| 431 | 27.3 | 14.7 | 2.9 | 9.3 | 0.9 | 0.8 | 5.1 | 2.7 | 1.0 | 8.4 | 0.4 | 0.2 | 0.3 | 2.3 | 2.6 | 6.7 | 3.1 | 2.0 |
| 436 | 20.1 | 9.8 | 3.0 | 12.5 | 2.4 | 0.7 | 5.5 | 4.1 | 0.8 | 9.7 | 1.8 | 1.8 | 2.1 | 1.2 | 2.9 | 5.3 | 2.2 | 2.0 |
| 439 | 34.2 | 13.6 | 2.5 | 10.1 | 1.0 | 0.7 | 6.5 | 2.7 | 0.8 | 8.0 | 0.5 | 0.2 | 0.3 | 1.5 | 1.9 | 5.4 | 2.8 | 1.3 |
| 442 | 17.8 | 8.7 | 2.2 | 9.0 | 2.7 | 0.6 | 5.3 | 4.1 | 0.7 | 11.6 | 2.0 | 1.5 | 2.0 | 0.8 | 6.7 | 11.9 | 2.7 | 3.2 |
| 444 | 16.8 | 8.5 | 2.1 | 8.5 | 2.8 | 0.5 | 6.0 | 4.2 | 0.6 | 9.3 | 2.5 | 2.2 | 2.9 | 0.6 | 3.1 | 8.6 | 2.9 | 2.1 |
| 446 | 28.0 | 17.5 | 3.8 | 8.9 | 0.8 | 1.2 | 5.5 | 3.0 | 1.3 | 7.6 | 0.3 | 0.1 | 0.2 | 1.6 | 1.7 | 5.6 | 5.2 | 1.4 |
| 449 | 19.9 | 14.7 | 3.3 | 9.3 | 2.1 | 0.9 | 4.7 | 4.8 | 1.0 | 7.5 | 1.6 | 1.1 | 1.6 | 0.9 | 1.8 | 5.2 | 3.7 | 1.2 |
| 451 | 19.2 | 13.6 | 2.7 | 7.6 | 1.8 | 0.8 | 4.2 | 3.8 | 0.8 | 7.2 | 1.5 | 1.0 | 1.5 | 0.7 | 2.3 | 7.2 | 2.7 | 1.3 |
| 456 | 19.0 | 12.1 | 2.4 | 9.3 | 1.5 | 0.6 | 5.3 | 2.9 | 0.7 | 8.1 | 0.9 | 0.7 | 0.8 | 0.7 | 2.0 | 7.9 | 3.4 | 1.6 |
| 458 | 18.8 | 15.2 | 5.2 | 5.0 | 2.4 | 1.5 | 3.3 | 7.2 | 2.3 | 5.2 | 1.7 | 1.2 | 1.8 | 0.6 | 1.6 | 5.1 | 4.2 | 0.9 |
| 460 | 18.2 | 16.3 | 4.8 | 8.2 | 0.7 | 1.8 | 1.6 | 4.6 | 1.9 | 6.4 | 0.7 | 0.9 | 1.0 | 1.6 | 1.1 | 2.7 | 3.5 | 0.7 |
| 463 | 17.9 | 15.8 | 3.8 | 7.0 | 1.0 | 1.1 | 2.4 | 3.9 | 1.4 | 6.3 | 0.9 | 0.5 | 0.9 | 1.1 | 1.8 | 6.3 | 3.4 | 0.9 |
| 466 | 14.7 | 11.5 | 2.7 | 7.3 | 1.7 | 0.6 | 4.1 | 3.9 | 0.7 | 6.5 | 1.3 | 0.9 | 1.2 | 0.7 | 1.8 | 6.9 | 3.8 | 0.9 |
| 470 | 21.8 | 19.1 | 4.0 | 6.2 | 0.9 | 1.3 | 5.3 | 4.3 | 1.2 | 6.1 | 1.3 | 1.4 | 1.3 | 1.1 | 1.4 | 5.2 | 5.3 | 1.2 |
| 472 | 15.8 | 19.2 | 6.4 | 4.8 | 1.3 | 1.6 | 2.1 | 4.7 | 2.2 | 6.3 | 1.1 | 0.9 | 1.3 | 2.0 | 2.1 | 7.5 | 7.5 | 0.8 |
| 477 | 17.6 | 20.2 | 5.9 | 4.4 | 1.4 | 1.7 | 2.7 | 5.3 | 2.3 | 4.8 | 1.5 | 1.4 | 1.8 | 1.6 | 1.3 | 5.2 | 6.9 | 0.7 |
| 479 | 14.7 | 20.8 | 7.7 | 3.5 | 1.2 | 1.9 | 2.0 | 4.9 | 2.7 | 4.6 | 1.1 | 0.9 | 1.4 | 2.4 | 1.5 | 5.4 | 11.0 | 0.6 |
| 481 | 20.5 | 27.8 | 7.6 | 5.2 | 0.4 | 2.4 | 1.7 | 2.1 | 2.9 | 4.2 | 0.5 | 0.5 | 0.6 | 2.7 | 0.8 | 3.3 | 6.8 | 0.5 |
| 485 | 18.2 | 28.0 | 9.1 | 4.7 | 0.3 | 2.4 | 2.1 | 1.4 | 2.7 | 4.2 | 0.3 | 0.2 | 0.3 | 3.7 | 0.9 | 3.6 | 8.1 | 0.5 |
| 488 | 16.6 | 21.3 | 7.5 | 3.4 | 0.9 | 2.0 | 2.3 | 4.2 | 2.9 | 3.4 | 1.0 | 0.9 | 1.4 | 2.8 | 1.0 | 3.6 | 10.0 | 0.6 |
| 491 | 18.9 | 26.7 | 7.1 | 3.8 | 0.4 | 2.6 | 1.8 | 1.8 | 3.3 | 3.7 | 0.5 | 0.5 | 0.6 | 4.7 | 0.9 | 3.7 | 7.1 | 0.5 |
| 495 | 27.3 | 27.0 | 5.9 | 3.4 | 0.6 | 2.4 | 2.2 | 2.4 | 3.1 | 2.4 | 0.6 | 0.6 | 0.7 | 2.0 | 0.6 | 1.7 | 4.2 | 0.5 |
| 502 | 29.1 | 26.1 | 6.9 | 3.4 | 0.5 | 2.4 | 2.0 | 1.7 | 3.1 | 3.3 | 0.4 | 0.6 | 0.6 | 2.6 | 0.7 | 3.2 | 4.4 | 0.6 |
| 505 | 29.5 | 27.3 | 6.2 | 3.4 | 0.3 | 2.1 | 2.1 | 1.5 | 2.4 | 2.6 | 0.2 | 0.2 | 0.2 | 3.5 | 0.6 | 2.0 | 4.9 | 0.6 |
| 507 | 26.8 | 25.1 | 6.9 | 3.3 | 0.4 | 2.1 | 2.4 | 1.7 | 2.4 | 2.8 | 0.4 | 0.4 | 0.4 | 3.3 | 0.7 | 2.6 | 5.2 | 0.6 |
| 508 | 19.7 | 21.1 | 6.5 | 3.8 | 0.8 | 1.9 | 2.1 | 3.5 | 2.5 | 4.2 | 1.3 | 1.4 | 1.6 | 2.7 | 1.2 | 4.8 | 5.4 | 0.6 |
| 509 | 31.1 | 25.1 | 5.4 | 3.3 | 0.4 | 1.8 | 2.9 | 1.6 | 2.1 | 2.7 | 0.3 | 0.3 | 0.3 | 3.1 | 0.6 | 2.4 | 4.8 | 0.7 |
| 512 | 30.9 | 26.5 | 5.4 | 3.4 | 0.3 | 1.8 | 1.9 | 1.4 | 2.1 | 2.8 | 0.3 | 0.2 | 0.3 | 3.7 | 0.6 | 2.4 | 4.4 | 0.6 |
| 514 | 34.7 | 26.3 | 4.8 | 3.5 | 0.2 | 1.9 | 1.9 | 1.3 | 2.0 | 3.2 | 0.3 | 0.3 | 0.4 | 4.6 | 0.8 | 3.3 | 5.8 | 0.5 |
| 516 | 35.5 | 27.8 | 5.0 | 3.2 | 0.2 | 1.7 | 1.6 | 1.3 | 1.8 | 2.7 | 0.3 | 0.3 | 0.3 | 3.3 | 0.6 | 2.3 | 4.7 | 0.4 |
| 519 | 37.2 | 25.2 | 5.4 | 2.7 | 0.3 | 1.7 | 2.9 | 1.6 | 2.0 | 2.0 | 0.2 | 0.2 | 0.2 | 3.0 | 0.5 | 1.6 | 4.0 | 0.5 |
| 521 | 35.1 | 23.2 | 5.0 | 3.5 | 0.4 | 1.6 | 2.0 | 1.9 | 1.9 | 3.2 | 0.4 | 0.4 | 0.5 | 3.5 | 0.8 | 3.5 | 5.4 | 0.6 |
| 523 | 36.9 | 24.0 | 5.2 | 5.4 | 0.3 | 1.7 | 1.6 | 1.9 | 1.7 | 3.8 | 0.3 | 0.8 | 0.5 | 4.4 | 0.8 | 1.7 | 5.3 | 0.6 |
| 526 | 39.8 | 25.0 | 4.1 | 3.0 | 0.3 | 1.4 | 2.4 | 1.5 | 1.6 | 2.6 | 0.2 | 0.2 | 0.2 | 2.4 | 0.7 | 2.6 | 3.4 | 0.5 |
