# Supplementary material for: Long-Term Biogas Production from Glycolate by Diverse and Highly Dynamic Communities
Source: Microorganisms. 2018 Oct 4;6(4):103. doi: 10.3390/microorganisms6040103 (PMC6313629; doi:10.3390/microorganisms6040103)
Supplement: Supplementary file 1 [file microorganisms-06-00103-s001.zip › microorganisms-356095-suppl-proofreading done/S4.pdf]

Table S4. Sorted sampling days and gates

Day	Gates	Relative abundance on sampled day [%]
495	G1	27.3
	G2	27
	G26	4.2
508	G25	2.4
	G3	5.4
	G8	2.9
	G6	1.8
