# Supplementary material for: Long-Term Biogas Production from Glycolate by Diverse and Highly Dynamic Communities
Source: Microorganisms. 2018 Oct 4;6(4):103. doi: 10.3390/microorganisms6040103 (PMC6313629; doi:10.3390/microorganisms6040103)
Supplement: Supplementary file 1 [file microorganisms-06-00103-s001.zip › microorganisms-356095-suppl-proofreading done/S5.pdf]

Supplemental Material S5. DNA extraction and library preparation for Illumina MiSeq sequencing

DNA extraction was performed according to Koch et al. (2013). 70 µL 10% (w/v) Chelex 100 solution (Bio-Rad) was used to extract DNA from 500,000 cells. The samples were heated at 90°C for 45 min and centrifuged for 5 min at 7,000 x g and 4°C before 50 µL of the supernatant containing the purified DNA was stored at -20°C. The quality of the extracted DNA was tested by a 35 cycle PCR with primers 27F (5'-AGA GTT TGA TCM TGG CTC AG-3') and 1492R (5'-TAC GGY TAC CTT GTT ACG ACT T-3') targeting bacterial 16S rRNA genes (Lane 1991).
For amplicon sequencing, the primers Pro341F (5'-CCT ACG GGN BGC ASC AG-3') and Pro805r (5'-GAC TAC NVG GGT ATC TAA TCC-3') were used for amplification of the V3-V4 region of the 16S rRNA gene while the primers 5'-GGT GGT GTM GGA TTC ACA CAR TAY GCW ACA GC-3' and 5'-TTC ATT GCR TAG TTW GGR TAG TT-3' were used to amplify the *mcrA* gene. The amplicon length was around 460-490 bp for both genes. After a 20 cycle PCR with the primers mentioned above, ten further PCR cycles were performed with barcoded primers. The PCRs were performed in 10 µL batches, containing 10 pmol of each primer (Eurofins), 2 nmol dNTP mix (Promega), 2 µL 5x Phusion® GC solution and 20 nmol $MgCl_2$, (both provided in the polymerase kit), 0.2 units Phusion® High-Fidelity Polymerase (New England Biolabs) and 1 µL DNA. Nuclease free water (Qiagen) was added and adjusted the final reaction volume. The PCR temperature protocol was as follows: initial denaturation for 3 min at 95°C, cycling of 95°C for 30 s, 55°C for 60 s, 72°C for 1 min, followed by a final extension at 72°C for 10 min before cooling down to 4°C. For each reaction, a no-template control was run for 35 cycles to ensure that no contamination was present. The PCR was done in triplicates for each sample. The triplicates were combined in equimolar proportion before the final pooling. All PCR runs were performed using a S1000 Thermal cycler (Bio-Rad).
The PCR products were purified with the Agencourt® AMPure® XP-Kit (Beckman Coulter Genomics) between and after the two steps. They were quantified with Qubit 3.0 (Life Technologies) using the Qubit dsDNA HS Assay Kit and finally pooled to 30 µL in equimolar proportion to be sequenced. Sequencing of the library was performed on the MiSeq platform (Illumina) using the 2 x 300 bp, 600 cycles option with the v3 kit (Illumina).

References:
Koch, C., Günther, S., Desta, A.F., Hübschmann, T. & Müller, S. (2013) Cytometric fingerprinting for analysing microbial intra-community structure variation and identifying sub-community function. Nature Protocols, 8, 190-202
Lane, D.J. (1991) 16S/23S rRNA sequencing. Nucleic Acids Techniques in Bacterial Systematics (eds Stackebrandt, E. & Goodfellow, M.), pp. 115-147. John Wiley & Sons, Chichester
