# Supplementary material for: Long-Term Biogas Production from Glycolate by Diverse and Highly Dynamic Communities
Source: Microorganisms. 2018 Oct 4;6(4):103. doi: 10.3390/microorganisms6040103 (PMC6313629; doi:10.3390/microorganisms6040103)
Supplement: Supplementary file 1 [file microorganisms-06-00103-s001.zip › microorganisms-356095-suppl-proofreading done/S6.pdf]

Table S6. Euclidian distances of dominant gates shown in Figure 3.

	G1	G2	G3	G4	G5	G6	G8	G9	G10	G15	G16	G17	G20	G21	G22	G25	G26
G2	8.58																
G3	10.56	3.85															
G4	16.18	21.90	22.42														
G5	19.97	24.01	24.02	13.07													
G6	12.89	5.42	4.20	25.23	26.44												
G8	13.07	18.82	19.57	10.65	15.45	22.10											
G9	12.23	14.11	13.40	14.04	14.28	16.10	13.71										
G10	14.34	7.20	5.24	26.10	27.12	3.01	23.22	16.38									
G15	12.99	18.57	19.25	5.46	13.06	22.02	9.47	11.79	23.09								
G16	17.12	20.83	20.62	12.45	9.85	23.31	13.79	9.64	23.90	11.54							
G17	18.38	22.75	22.71	12.07	12.10	25.27	14.19	12.80	26.02	12.35	6.27						
G20	16.45	19.66	19.39	13.63	10.84	22.14	14.22	9.69	22.83	12.11	3.40	7.21					
G21	9.97	10.92	12.85	21.05	24.39	13.19	18.27	17.78	14.96	18.10	21.71	22.35	20.94				
G22	12.94	18.09	18.62	10.07	14.65	21.11	8.24	11.86	22.29	6.45	11.88	12.60	12.23	17.78			
G25	12.25	17.85	18.62	8.97	15.11	21.09	8.48	12.31	22.28	5.30	12.61	12.98	13.12	16.66	4.28		
G26	7.56	7.24	8.13	19.27	21.59	10.05	15.56	12.80	11.31	15.74	18.51	20.27	17.47	9.42	14.96	14.59	
G27	14.42	20.10	20.89	13.27	17.17	22.99	8.07	15.39	24.34	10.90	14.29	14.24	14.56	18.21	6.46	7.98	16.77
