# Supplementary material for: Long-Term Biogas Production from Glycolate by Diverse and Highly Dynamic Communities
Source: Microorganisms. 2018 Oct 4;6(4):103. doi: 10.3390/microorganisms6040103 (PMC6313629; doi:10.3390/microorganisms6040103)
Supplement: Supplementary file 1 [file microorganisms-06-00103-s001.zip › microorganisms-356095-suppl-proofreading done/S7_SK.docx]

**Table S7.** Cluster abundances per phase. Sum of average gate abundances (see Table S3a) in cluster 1 (G1, G2, G3, G6, G10, G21, G26), cluster 2 (G4, G8, G15, G22, G25, G27), and cluster 3 (G5, G9, G16, G17, G20) for each phase (P1-P6).

| **Phase** | **P1** | **P2** | **P3** | **P4** | **P5** | **P6** | **All days** |
| --- | --- | --- | --- | --- | --- | --- | --- |
| Days | 1-180 | 183-232 | 234-281 | 284-414 | 416-470 | 472-526 | 1-526 |
|  | Sum of mean abundances [%] | Sum of mean abundances [%] | Sum of mean abundances [%] | Sum of mean abundances [%] | Sum of mean abundances [%] | Sum of mean abundances [%] | Sum of mean abundances [%] |
| Cluster 1 | 38.90 | 55.99 | 56.00 | 71.61 | 44.84 | 71.09 | 55.29 |
| Cluster 2 | 29.70 | 20.39 | 25.23 | 12.26 | 26.92 | 13.97 | 21.66 |
| Cluster 3 | 8.23 | 6.31 | 6.63 | 5.04 | 11.33 | 4.73 | 6.98 |
